# Classification of benign and malignant subtypes of breast cancer histopathology imaging using hybrid CNN-LSTM based transfer learning

**DOI:** 10.1186/s12880-023-00964-0

**Published:** 2023-01-30

**Authors:** Mahati Munikoti Srikantamurthy, V. P. Subramanyam Rallabandi, Dawood Babu Dudekula, Sathishkumar Natarajan, Junhyung Park

**Affiliations:** 1 3BIGS Omicscore Pvt. Ltd., 909 Lavelle Building, Richmond Circle, Bangalore, 560025 India; 2 3BIGS Co. Ltd, 156, B-831, Geumgang Penterium IX Tower, Hwaseong, 18469 Republic of Korea

**Keywords:** Histopathology, Breast cancer, Deep learning, Image classification

## Abstract

**Background:**

Grading of cancer histopathology slides requires more pathologists and expert clinicians as well as it is time consuming to look manually into whole-slide images. Hence, an automated classification of histopathological breast cancer sub-type is useful for clinical diagnosis and therapeutic responses. Recent deep learning methods for medical image analysis suggest the utility of automated radiologic imaging classification for relating disease characteristics or diagnosis and patient stratification.

**Methods:**

To develop a hybrid model using the convolutional neural network (CNN) and the long short-term memory recurrent neural network (LSTM RNN) to classify four benign and four malignant breast cancer subtypes. The proposed CNN-LSTM leveraging on ImageNet uses a transfer learning approach in classifying and predicting four subtypes of each. The proposed model was evaluated on the BreakHis dataset comprises 2480 benign and 5429 malignant cancer images acquired at magnifications of 40×, 100×, 200× and 400×.

**Results:**

The proposed hybrid CNN-LSTM model was compared with the existing CNN models used for breast histopathological image classification such as VGG-16, ResNet50, and Inception models. All the models were built using three different optimizers such as adaptive moment estimator (Adam), root mean square propagation (RMSProp), and stochastic gradient descent (SGD) optimizers by varying numbers of epochs. From the results, we noticed that the Adam optimizer was the best optimizer with maximum accuracy and minimum model loss for both the training and validation sets. The proposed hybrid CNN-LSTM model showed the highest overall accuracy of 99% for binary classification of benign and malignant cancer, and, whereas, 92.5% for multi-class classifier of benign and malignant cancer subtypes, respectively.

**Conclusion:**

To conclude, the proposed transfer learning approach outperformed the state-of-the-art machine and deep learning models in classifying benign and malignant cancer subtypes. The proposed method is feasible in classification of other cancers as well as diseases.

**Supplementary Information:**

The online version contains supplementary material available at 10.1186/s12880-023-00964-0.

## Background

Breast cancer is the most common cancer affecting particularly women. In 2020, there were about 2.3 million breast cancer cases diagnosed including 685 000 deaths worldwide. By 2020, there were 7.8 million women alive in the past 5 years and became the world’s most dominant cancer [1]. The need for accurate and automated clinical diagnosis with the help of machine learning (ML) based histopathological grading that will lead to effective treatments. Although the survival rates of breast cancer are drastically improving, still there is a lack of awareness, and screening procedures along with a shortage of medical imaging facilities. Hence, the survival rates of breast cancer were found to be above 80%, 60% and below 40% in developed countries, developing countries, and low-income or underdeveloped countries, respectively.

Mammography and ultrasound imaging are the common imaging modalities that are used to detect cancers with the help of radiologists. However, for grading of cancer, pathologists and expert clinicians need to manually visualize whole-slide images which is a cumbersome task, time-consuming and may also lead to wrong decisions based on the different diagnostic criteria available globally. Hence, an automated computer-aided decision (CAD) support system will help in reducing false positives and improve diagnostic accuracy with less expert clinician intervention. The developed ML models require some domain knowledge as it needs feature extraction and feature selection processes to build the models. However, the development of deep learning (DL) models tremendously reduces the feature extraction and feature engineering processes by using the applicability of convolutional neural networks (CNN) such as ConvNet [2], ImageNet [3], and DenseNet [4].

Different CNN models like VGG16, VGG19, InceptionV3, and ResNet50 were compared in classifying benign and malignant breast cancer images [5]. A CNN model was proposed for the feature extraction of breast cancer histopathological images and classification using support vector machines (SVM) [6]. Recently, a novel deep neural network was proposed using the clustering method and CNN model, Long-Short-Term-Memory (LSTM), and a combination of CNN and LSTM models [7]. A DL framework that can learn features automatically from mammography images [8], AlexNet as a feature extractor and applied SVM as a classification model [9], and, a hybrid CNN model using AlexNet, MobileNetV2 and ResNet50 [10].

Various approaches have been proposed for the classification of histopathological images using a nucleus-guided feature extraction framework based on CNN approach [11], and, automated segmentation of glandular epithelium on haematoxylin and eosin (H&E), and immunohistology compatibility (IHC) stain images [12]. Another study proposed a weak supervised learning approach using multiple instance learning (MIL) and compared several MIL methods such as Axis-Parallel Rectangle (APR), diverse density, MIL-support vector machines, *k*-nearest neighbour; and reported that non-parametric approach using MIL-CNN deep learning model was outperformed the other methods [13]. A DL-based CNN approach was proposed to automatically segment and classify the epithelial and stromal regions from the microarrays of digital tumor tissue. Most of the approaches are based on low-level image features, such as color, texture, and local binary patterns (LBP) in classifying two regions. The deep CNN feature extractor is directly learned from the raw pixel intensity value of epithelial and stromal tissues unlike the low-level image feature-based approaches, which involve task dependent representation [14]. Another study proposed a segmentation method to delineate cells using gaussian-based hierarchical voting and repulsive balloon model and classify adenocarcinoma and squamous carcinoma [15]. Al-Kadi used four texture features (two statistical features and two model-based) and noticed that the combined Gaussian Markov random field and run-length matrix texture measures using the Bayesian classifier outperformed in classifying meningioma tissue [16]. The authors compared the performance of different pre-trained models such as VGG16, Inception [17], ResNet, and NASNet [18] using a transfer learning approach and the combined dataset the NASNet achieved significant accuracy.

Here in this paper, we propose a hybrid CNN, long short-term memory recurrent neural network (CNN-LSTM) model leveraging on ImageNet weights using a transfer learning approach in classifying the subtypes of benign and malignant breast cancer histopathological images. Further, we discuss the methodology of the proposed hybrid CNN-LSTM model including the details of the dataset used for validation of our proposed method in classifying the subtypes of benign and malignant breast cancer histopathological images. In the following section, we report the results using the proposed approach and compared them with existing methods. Later, we discuss the advantages and limitations of the proposed approach over existing methodologies and finally, we conclude the study.

## Methods

### Dataset

The Breast Cancer Histopathological Image Classification (BreakHis) [19] is a public dataset composed of 7909 microscopic images of breast tumor tissue collected from 82 patients using different magnifying factors (40×, 100×, 200×, and 400×). It contains 2480 benign and 5429 malignant samples (700 × 460 pixels, 3-channel RGB, 8-bit depth in each channel, PNG format). This database has been built in collaboration with the P&D Laboratory - Pathological Anatomy and Cytopathology, Parana, Brazil [19]. The more details about different classifications along with four distinct benign subtypes adenosis (A), fibroadenoma (F), phyllodes tumor (PT), and tubular adenoma (TA), malignant subtypes ductal carcinoma (DC), lobular carcinoma (LC), mucinous carcinoma (MC) and papillary carcinoma (PC) are given in Table 1.

### Data augmentation

In order to avoid overfitting and unbalanced class problems, we perform multi-scale data augmentation for the training dataset by a random combination of intensity variation, rotation, flip with horizontal and vertical direction, and translation. The data augmentation was performed using Keras built-in function ImageDataGenerator. The images were flipped horizontally and vertically, rotated ± 20 degrees, featurewise center was set to ‘True’. The classes of breast cancer are imbalanced due to a large amount of ductal carcinoma, which shows the Gaussian distribution, we used an over-sampling method using above mentioned data augmentation approaches to balance the number of breast cancer histopathological images of each class.

**Table 1:** Classification of images based on tumor subtypes along with the magnification factors

Classes	Subclasses	Number of patients	Magnification factors				Total
			40×	100×	200×	400×	
Benign	Adenosis (class 0)	4	114	113	111	106	444
	Fibroadenoma (class 1)	10	253	260	264	237	1014
	Tubular adenoma (class 2)	3	109	121	108	115	453
	Phyllodes adenoma (class 3)	7	149	150	140	130	569
Malignant	Ductal carcinoma (class 4)	38	864	903	896	788	3451
	Lobular carcinoma (class 5)	5	156	170	163	137	626
	Mucinous carcinoma (class 6)	9	205	222	196	169	792
	Papillary carcinoma (class 7)	6	145	142	135	138	560
Total		82	1995	2081	2013	1820	7909

### Proposed CNN-LSTM model

**Fig. 1:**
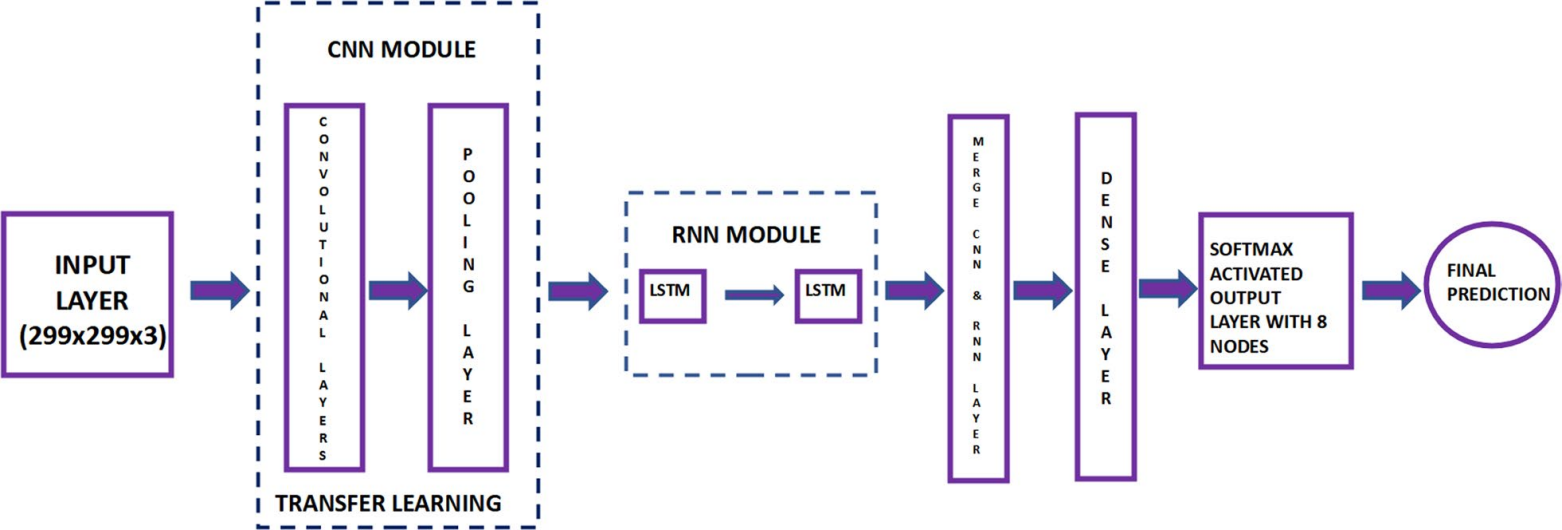
The schema of the proposed deep learning approach

**Fig. 2:**
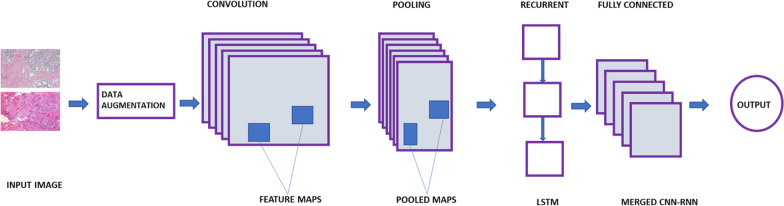
The architecture of the proposed hybrid CNN-LSTM model

The proposed hybrid CNN-LSTM model mainly consists of two modules, the CNN with (299 × 299 × 3) input shape and an independent RNN module. The CNN is passed through a pre-trained transfer learning model (InceptionResNetV2, ResNet50) until it reaches the final convolutional layer which has the bottleneck features of size (batch size,2048), whereas, the independent RNN module has 2 LSTM layers each of which are of (batch size, 2048). The outputs of both modules are merged using element-wise multiplication. This output is fed into the classification layer of 8 nodes (8 classes) and SoftMax activation function. Since each convolutional layer can capture images of a fixed length, convolutional layers of different filter lengths can detect images of magnification factor. The proposed model mainly contains a convolutional module, a pooling module, LSTM dense layer, and a fully connected module including a SoftMax activation functions as given in Fig. 1. Figure 2 shows the architecture of the proposed hybrid CNN-LSTM model.

### Model optimization

The hyperparameters were used for training and optimizing or finetuning the DL models. For initial training and finetuning, we used model check pointers and callbacks monitoring the loss to prevent overfitting, and, the weights of the best model were saved. For the final production model of each magnification factor, both binary classification and the multi-class classification of benign, and malignant subtypes, different optimizers, learning rate, varying epochs and hyperparameters with tolerance 1e−3 were chosen such that the model should not overfit.

The fine-tuned model using these optimal parameters was finally tested for the prediction of the benign and malignant stages. The parameters used in this work are reported in Table 2.

**Table 2:** Model parameters for pre-trained CNN model architecture

Model parameters	Values
No. of filter (conv 1)	64
Pooling window size (avg 1)	2
LSTM layers	2
Batch size	32 and 16
Batch normalization (yes/no)	Yes
Activation function	SOFTMAX
Optimizer	ADAM
Learning rate	0.001
No. of epochs	500[Binary Classification] and 300[Multiclass Classification]

### Run environment and implementation

**Fig. 3:**
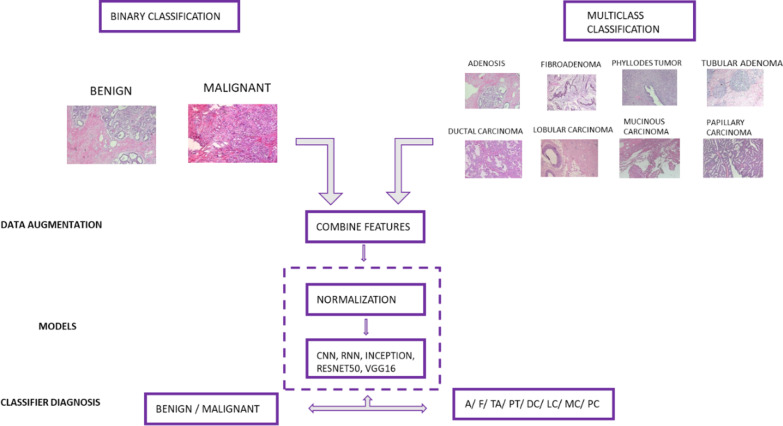
The workflow of the proposed deep learning approach

The hardware used in the execution of the DL models using Google Collaboratory with 52GB of RAM, NVidia GeForce GPU and development tools on the Ubuntu 64-bit operating system using Python 3.7.13. In addition, keras 2.8, TensorFlow 2.8.0, CUDA 10.0, cuDNN 7.6.5 libraries were used for DL. Matplotlib and Seaborn packages were used for visualization. Figure 3 shows the schematic illustration for the classification of benign and malignant breast cancer images using a hybrid CNN-LSTM DL model.

## Results

We compared the outcomes of the proposed model with the existing state-of-the-art CNN models. After pre-processing data, data augmentation was applied for generating more image samples to train the model by performing translation, rotation, and scaling including color normalization, and, splitting the training and test sets. The generated training and test sets were validated through the leave-one-out (LOO) cross-validation strategy using root-mean-square error (RMSE) as the scoring function. In the learning process, the neural network model was repeatedly performed until the loss converges to 1e−03 to correct the weights for improving accuracy. Various pre-trained CNN model architectures such as ResNet50, Inception, and Inception-ResNetv2 were implemented with ImageNet model weights. Finally, based on the finetuned hybrid CNN-LSTM model, the prediction was performed with an untrained test set as input.

### Evaluation of prediction results

We have evaluated various performance measures for the classification of benign and malignant cancer images such as accuracy, precision, and recall for both binary and multi-class classifiers as shown in.

Tables 3 and 4, respectively by utilizing the confusion matrix with true positives (TP), false positives (FP), true negatives (TN), and false negatives (FN) as per Eqs. 1, 2, 3.

1 $${\text{Accuracy}} = {\text{ }}\left( {{\text{TP}} + {\text{TN}}} \right)/\left( {{\text{TP}} + {\text{TN}} + {\text{FP}} + {\text{FN}}} \right)$$

2 $${\text{Precision}} = {\text{TP}}/\left( {{\text{TP}} + {\text{FP}}} \right)$$

3 $${\text{Recall}} = {\text{TP}}/\left( {{\text{TP}} + {\text{FN}}} \right)$$

**Fig. 4:**
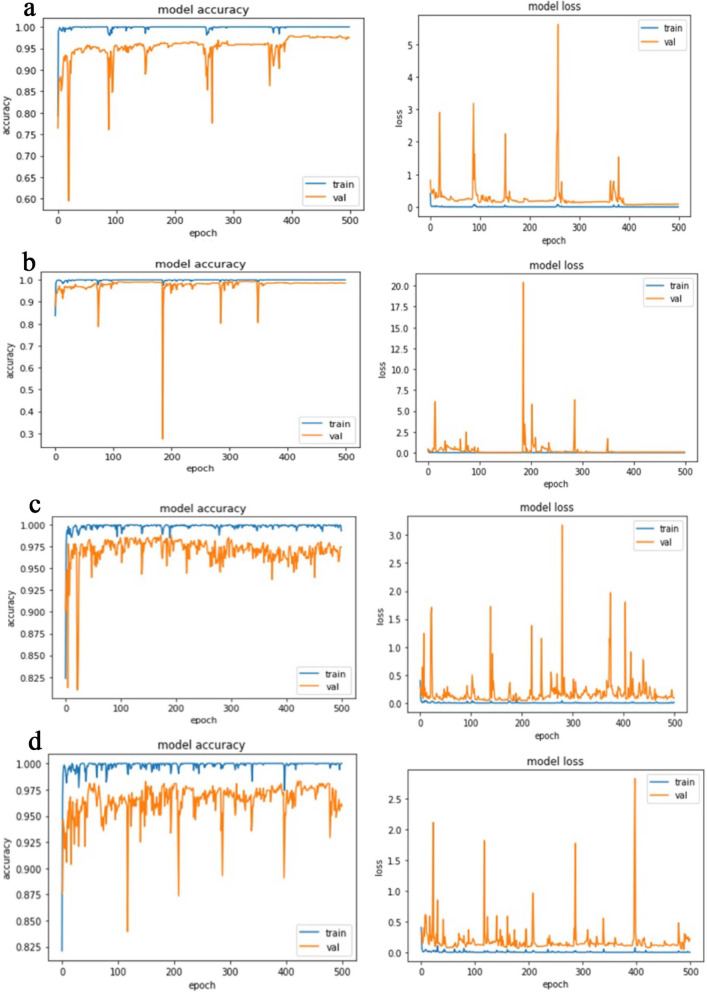
The binary classifier accuracy and error loss plots for **a** 40×, **b** 100×, **c** 200×, and **d** 400× panel using Adam optimizer

**Fig. 5:**
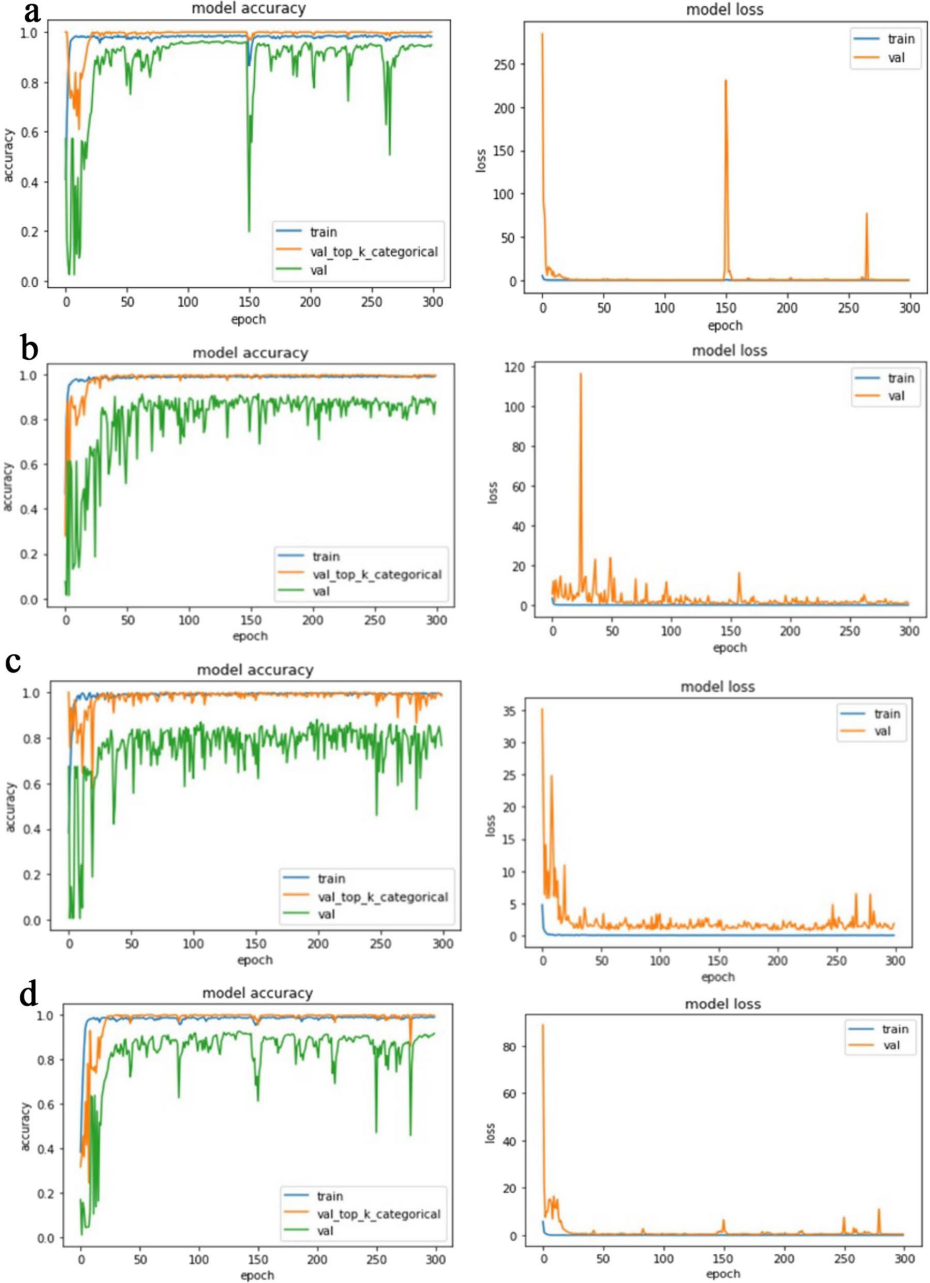
The multi-class classifier accuracy and error loss plots for **a** 40×, **b** 100×, **c** 200×, and **d** 400× panel using Adam optimizer

**Fig. 6:**
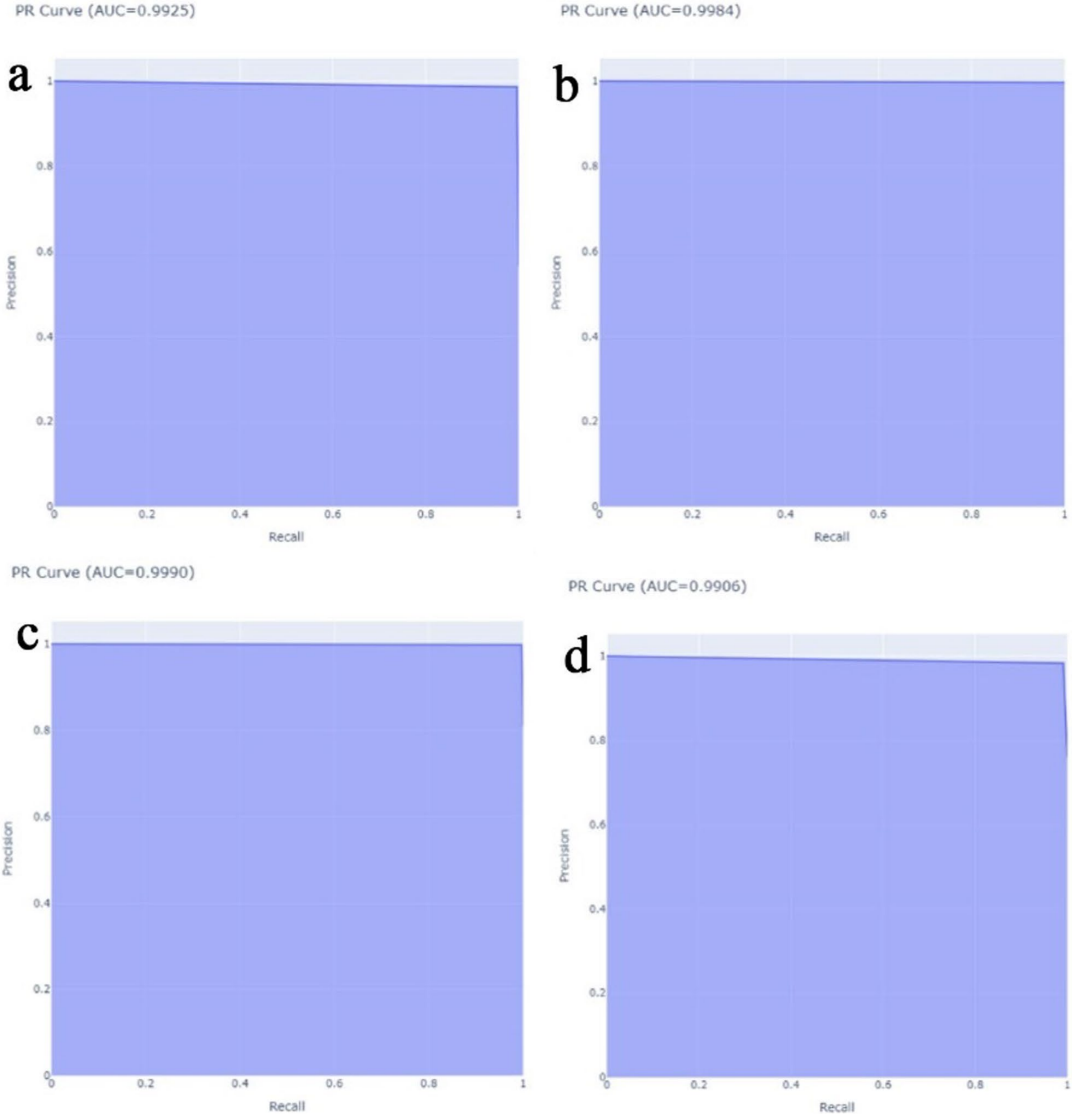
The binary classifier precision-recall curves for **a** 40× (top left), **b** 100× (top right), **c** 200× (bottom left), **d** 400× (bottom right) panel respectively using Adam optimizer

**Fig. 7:**
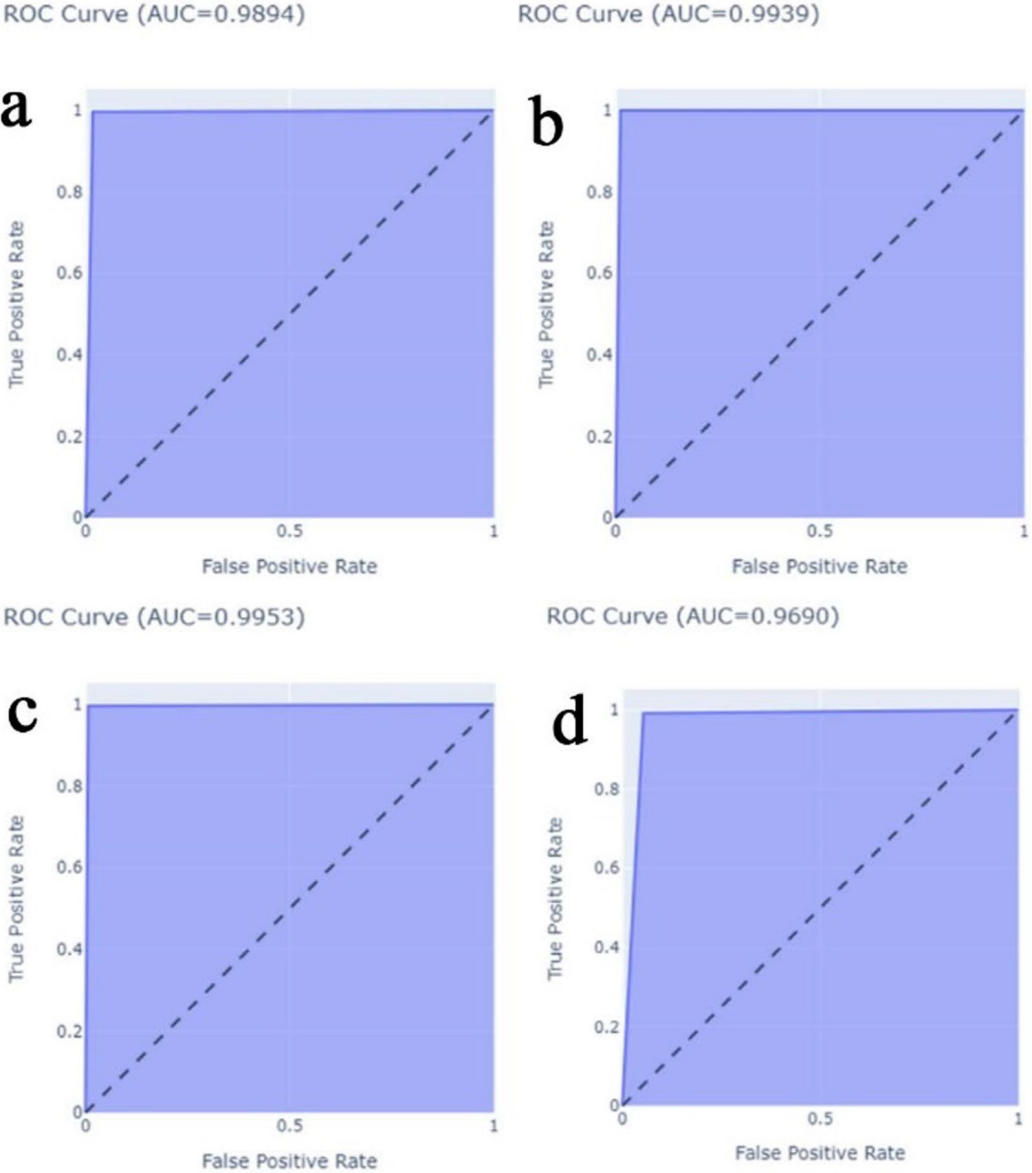
The binary classifier ROC curves for **a** 40× (top left), **b** 100× (top right), **c** 200× (bottom left), and **d** 400× (bottom right) panels respectively using Adam optimizer

**Fig. 8:**
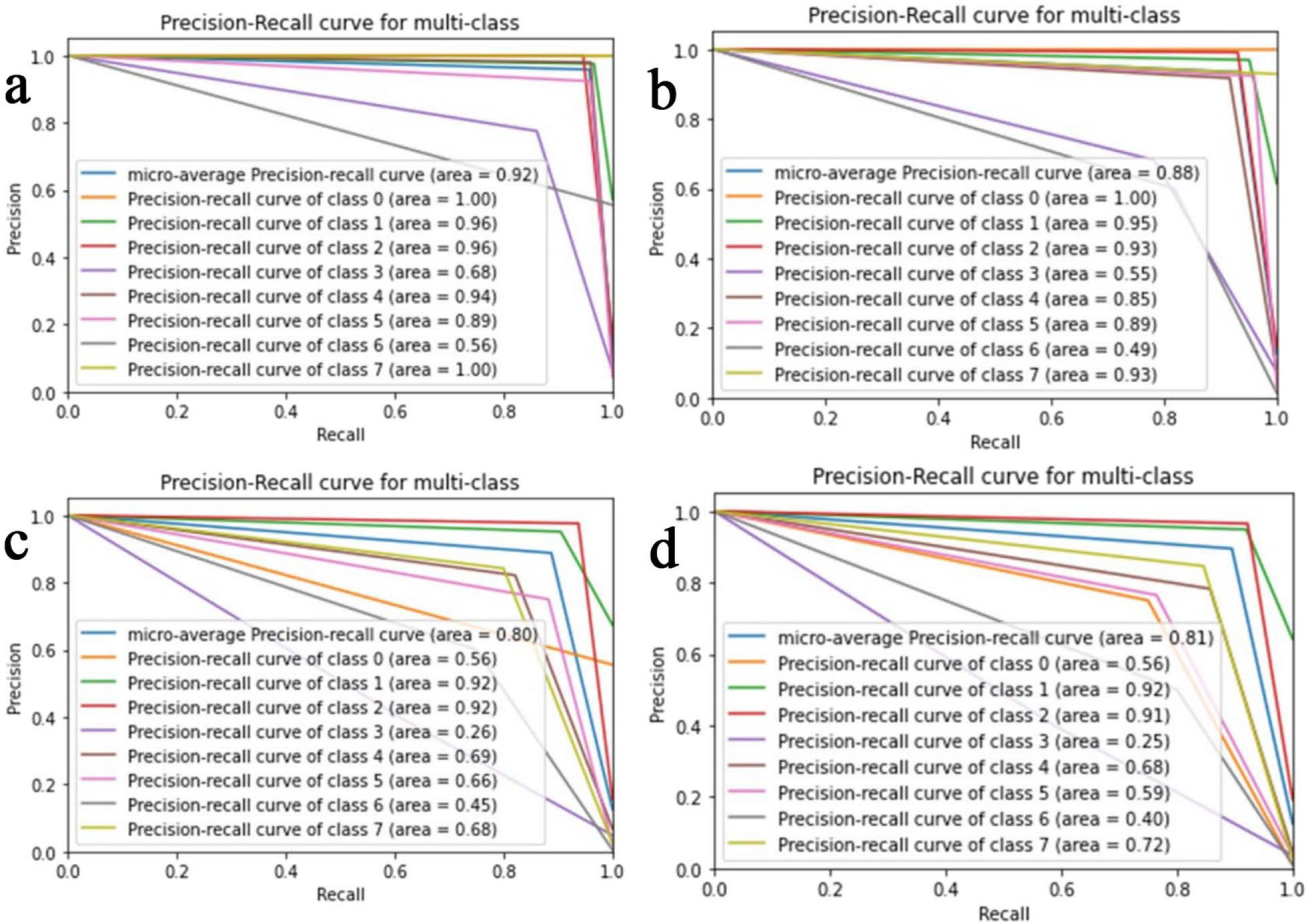
The multi-class classifier precision-recall curves for **a** 40× (top left), **b** 100× (top right), **c** 200× (bottom left), and **d** 400× (bottom right) panels respectively using Adam optimizer

**Fig. 9:**
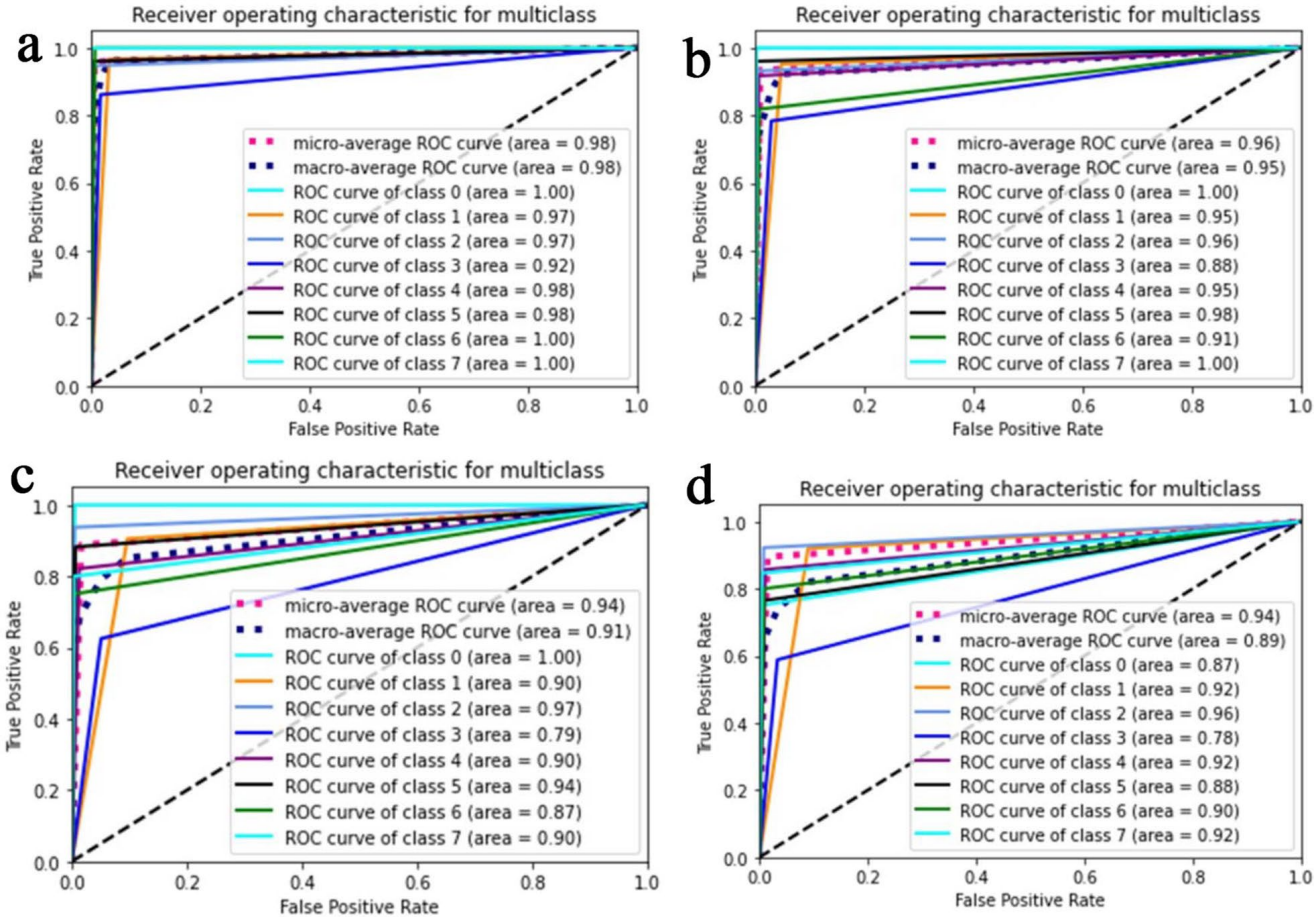
The multi-class classifier ROC curves for **a** 40× (top left), **b** 100× (top right), **c** 200× (bottom left), and **d** 400× (bottom right) panels respectively using Adam optimizer

**Fig. 10:**
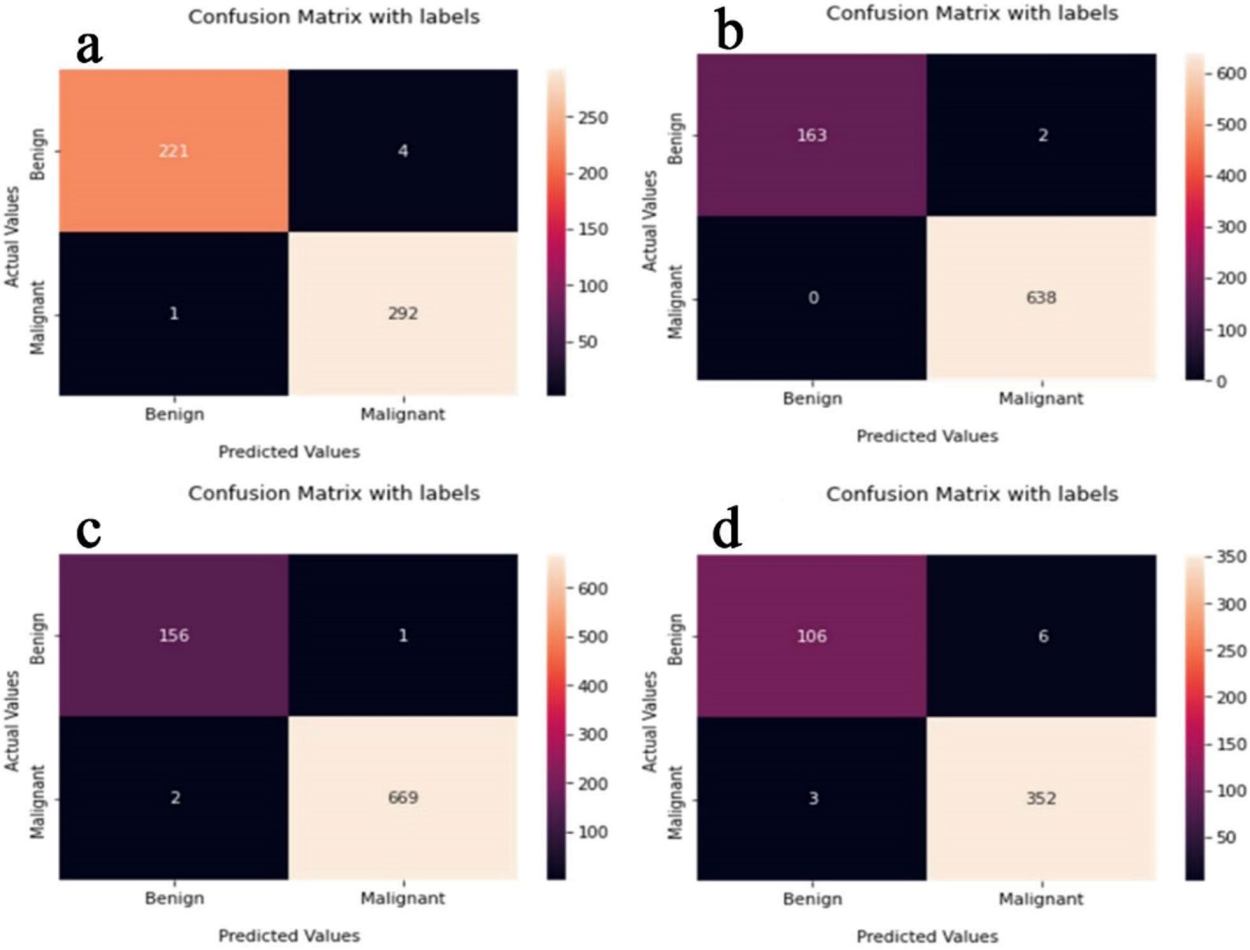
The binary classifier confusion matrix for **a** 40× (top left), **b** 100× (top right), **c** 200× (bottom left), and **d** 400× (bottom right) panels respectively using Adam optimizer

**Fig. 11:**
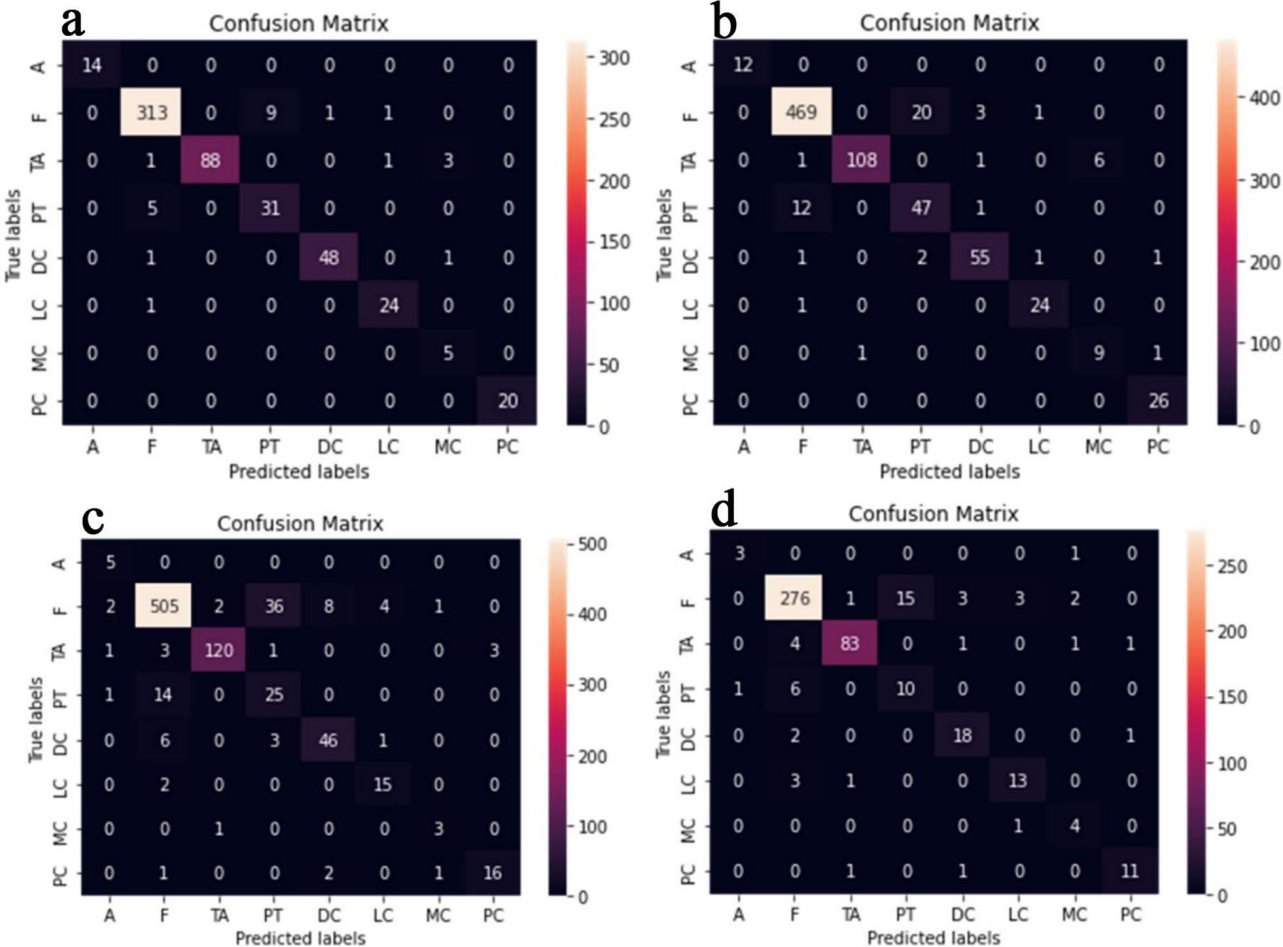
The multi-class classifier confusion matrix for **a** 40× (top left), **b** 100× (top right), **c** 200× (bottom left), **d** 400× (bottom right) panels respectively using Adam optimizer

The proposed CNN-LSTM binary classifier for 40×, 100×, 200× and 400× images showed an accuracy of 99.03, 99.75, 99.64, and 98.07% respectively as shown in Table 3. Accuracy and error plots of the same are given in Fig. 4a–d. The top *k*-categorical accuracy for multi-class classifiers were found to be 1.000, 0.998, 0.996 and 1.000 for 40×, 100×, 200× and 400× images respectively and these are given in Table 4. The proposed CNN-LSTM multi-class classifier for 40×, 100×, 200× and 400× images showed an accuracy of 96.5, 92.6, 88.04 and 92.51% respectively as shown in Table 4. The accuracy and error plots of the above are portrayed in Fig. 5a–d. We noticed that the proposed hybrid CNN-LSTM model outperformed the existing methods for both the binary and the multi-class classifiers as reported in Table 5. Figures 4a–d and 5a–d show binary and multi-class classification accuracy and error loss plots by varying the number of epochs for the training and validation sets using Adam (best optimizer) for different magnification factors 40×, 100×, 200× and 400× respectively. Figure 6a–d shows the binary classifier and Fig. 7a–d shows multi-class classifier precision-recall (PR) curves representing the tradeoff between precision (on y-axis) and recall (on x-axis) for various thresholds. The proposed CNN-LSTM binary classifier for 40×, 100×, 200× and 400× images showed the precision recall curve area under the curve (PR AUC) of 0.992, 0.998, 0.999 and 0.991 respectively as shown in Fig. 6a–d. The receiver-operating characteristics (ROC) curves by taking the false positive rate (on x-axis) and true positive rate (on y-axis) will also be taken as the performance measure. The proposed CNN-LSTM binary classifier for 40×, 100×, 200× and 400× images showed ROC AUC of 0.989, 0.994, 0.995 and 0.969, respectively as given in Fig. 7a–d. Figure 8a–d shows the average PR AUC of 0.92, 0.88, 0.8 and 0.81 for multi-class classifiers and Fig. 9a–d shows the ROC AUC of 0.98, 0.95, 0.91 and 0.89 for different magnification factors 40×, 100×, 200× and 400×, respectively. The confusion matrix for binary classifier 5 out of 518 samples, 2 out 803 samples, 3 of 828 samples, and 9 out of 467 samples were misclassified as shown in Fig. 10 for 40×, 100×, 200× and 400× respectively. Similarly, the confusion matrix for multi-class classifier 24 of 479 samples, 53 out of 803 samples, 93 out of 828 samples, 49 out of 467 samples were misclassified for 40×, 100×, 200× and 400× respectively as portrayed in Fig. 11. The results for the RMSprop and SGD optimizers using the proposed hybrid CNN LSTM model were provided in Additional file 1.

**Table 3:** Performance indices for binary classification of benign and malignant cancer

Magnification	Training: testing samples	Accuracy (%)	Precision	Recall
40×	1428:567	99.03	0.99	0.99
100×	1278:803	99.75	1.00	0.99
200×	1185:828	99.64	0.99	1.00
400×	1353:467	98.07	0.98	0.97

**Table 4:** Performance indices for multi-class classification of benign and malignant subtypes

Magnification	Training: testing samples	Accuracy (%)	Precision	Recall	Top K- categorical accuracy
40×	1428:567	96.3	0.97	0.95	1.00
100×	1278:803	92.6	0.92	0.93	0.9987
200×	1185:828	88.04	0.87	0.88	0.9963
400×	1353:467	92.51	0.92	0.93	1.00

**Table 5:** Comparison of proposed hybrid CNN-LSTM with existing state-of-the-art models

Study	Classifier	Transfer learning	Accuracy (%)			
			40×	100×	200×	400×
*Binary classification*						
Gupta et al. [20]	DR(DenseNet-169,XGB)	ImageNet	94.71 ± 0.88	95.9 ± 4.2	96.76 ± 1.09	89.11 ± 0.12
Nahid et al. [21]	NDCNN	None	94.4	95.93	97.19	96
Wei et al. [22]	NDCNN(GoogleNet)	ImageNet	97.89	97.64	97.56	97.97
Das et al. [23]	GoogleNet	ImageNet	94.82	94.38	94.67	93.49
Han et al. [24]	NDCNN(GoogleNet)	ImageNet	95.8 ± 3.1	96.9 ± 1.9	96.7 ± 2.0	94.9 ± 2.8
Gandomkar et al. [25]	ResNet-152	ImageNet	98.6	97.9	98.3	97
	Emdt(ResNet-152)	ImageNet	98.77 (overall)			
Bardou et al. [26]	Eiter(NDCNN)	None	98.33	97.12	97.85	96.15
Proposed model	CNN-RNN hybrid	ImageNet	99.03	99.75	99.64	98.07
*Multi-class classification*						
Han et al. [24]	NDCNN(GoogleNet)	ImageNet	92.8 ± 2.1	93.9 ± 1.9	93.7 ± 2.2	92.9 ± 1.8
Gandomkar et al. [25]	ResNet-152	Im-Break	95.6	94.8	95.6	94.6
Bardou et al. [26]	Eiter(NDCNN)	None	88.23	84.64	83.31	83.98
Nawaz et al. [27]	ResNet	ImageNet	95 (overall)			
Proposed model	CNN-RNN hybrid	ImageNet	96.5	92.6	88.94	92.51

## Discussion

Our proposed model achieved about 99% in binary classification (benign vs. malignant) and 92.50% for multi-class classifier in classifying subtypes of benign and malignant cancer when compared to the state-of-the-art methods that use four pre-trained models VGG16, VGG19, InceptionV3, and ResNet50 on a dataset that consists of 5000 breast images comprised of 2500 benign and 2500 malignant cases. The InceptionV3 model achieved the highest AUC of 0.91 [5]. Another CNN model using the local and frequency domain information showed an accuracy of 94.94% [6]. Also, the CNN model with gradient boosted tree classifier showed 87.20% accuracy for 4-class classification and for 2-class classification to detect breast carcinomas with an accuracy, sensitivity, specificity, and AUC of 93.80, 97.30, 96.50, 88.00%, and 0.973 respectively [28]. A recent study using HIC-Net showed an accuracy, sensitivity, and specificity of 96.21, 96.71 and 95.70% respectively [29].

There are several limitations of using less training data such as medical imaging datasets. To avoid this problem, data augmentation techniques will be used to train and validate more samples. A study was implemented with two different approaches using a CNN and a transfer learning model to classify breast cancer by combining two different datasets. The model showed better performance results combining traditional and generative adversarial networks (GAN) augmentation techniques [30]. Slide preparation and staining is another limitation as histopathological slides are scanned at different magnification factors using brightfield illumination, resulting in giga-pixel size for the entire image slice. Small square regions (called tiles or patches) are extracted and those that have a specified proportion of background pixels are removed from the dataset to overcome the high dimensionality of the image slices.

One of the major challenges for DL training data sets is the annotation of data and often carried out manually. Consider the ImageNet database, the images contained in this database were annotated through crowd-sourcing. Moreover, in domains like medical imaging, such annotations must be performed by professionals, which often increases the cost of such projects. The performance of DL models may be reduced as whole image slices contain both tumor and normal tissues [31]. A trained pathologist is required to delineate the tumor tissue in order to achieve tile level annotation. The whole image segmentation process comes with high cost and time which motivated the researchers to explore DL approaches to achieve automatic pixel level annotations from slide level labels. It has been reported that ensemble segmentation models with several fully connected convolutional networks showed a higher performance than a single neural network model [32]. Furthermore, tumor/normal tissue segmentation is required for the prediction of molecular genetics using DL, and molecular assays on large datasets are required to determine the correct labels for training data.

The main challenge is the requirement of the collection of huge data samples to train the models. The images collected across different scanners and research centers are integrated into a single dataset which can lead to bias and variance in the data. These variations in the dataset must be mitigated in order to avoid batch effects, bias reduction, and enhanced model performance [33]. For instance, biases between image slides might be due to the variation of light conditions while staining, concentrations and volumes of stain used in slide preparation. Also, different resolutions and magnification factor variability may further aggravate such biases. Recent studies also suggested that image slides preserve site-specific information which can be learned by a DL algorithm, resulting in overfitting of model performance [34]. Stain color normalization results to handle such batch effects by transforming pixel values from different image slides within a dataset to a common distribution (gaussian). On the other hand, color augmentation attempts to improve a model’s ability to generalize unseen data by simulating realistic color variations. Improved validation accuracies have been reported after applying color normalization and data augmentation such as scaling and rotation of the input images [35].

In order to encourage researchers to work on the DL models, ImageNet Large Scale Visual Recognition Challenge was conducted [36]. This challenge resulted in the development of various sophisticated and efficient DL models that proposed a variety of model architectures that have been re-modelled to a wide range of imaging applications such as computer vision, pattern recognition and digital pathology. In order to speed up the training processes, researchers can utilize domain transfer learning on the ImageNet model parameters These existing model parameters can be fixed, or in other terms, weights will not be updated during backpropagation. This approach is flexible in reducing the computational time and utilizing the model weights that have previously been shown in other image classification or pattern recognition tasks. For instance, all ImageNet layers except the final output layer may form the basis of a predictive model in a different domain and endure model parameters freezing with an extra randomly initialized SoftMax layer generating the output for the target domain. This allows the training process to finetune the weights of just one layer as opposed to a large and arbitrary number of layers. Transfer learning has the advantage of extenuating sample size requirements in the target domain by using existing model parameters trained on larger data sets. The domain transfer learning methods are most often used in digital pathology in order to avoid higher computational time as well as the cost involved in curating large training datasets. DL models in digital pathology utilize the majority voting strategy of aggregating predictions at the local region-level in order to obtain a per-slide or global image level prediction from the ML approaches [37]. DL can even detect microsatellite instability from the histopathological images and conditional generative adversarial networks (CGANs) retain information about genetic alterations [38].

The main advantages of the proposed method over existing methods are, firstly, feature extraction as well as feature dimensionality reduction is not required. Secondly, most of the medical imaging datasets have imbalanced class samples across different subtypes which may impact the prediction performance and bias towards the majority class. Hence, to avoid this issue, data augmentation was performed using random combinations of intensity variation, rotation, translation, horizontal and vertical flipping methods to avoid model overfitting or underfitting. Thirdly, the proposed model is feasible for applying to other diseases and scalable for other magnification images as well. Lastly, the proposed method can handle color histopathological images and can be helpful for the automated diagnosis of multi-class benign and malignant breast cancer subtypes with less intervention of a pathologist. However, there are a few limitations in our study. Firstly, the current model cannot predict the cancer stage progression or stability over time (as longitudinal data is required). Also, our current model is limited to 40×, 100×, 200× and 400× magnification images and however, newer models have to be built for other magnification images for further predictions. Another limitation, it is computationally expensive, complex, and tedious to implement on a normal workstation.

The major hindrance for the prevalent acceptance of DL systems in healthcare diagnostics is the lack of interpretability of data. Meaningful conclusions of DL predictions are critical in healthcare in order to take decisions from both a clinician’s and patient’s point of view for further clinical translation. DL models are mainly capable of handling the “black-box” model typically not showing the human-interpretable features that were selected in the model building. Furthermore, the DL models are capable of handling the noise or artifacts in the data being exploited by models to make predictions. DL models have already been developed using various methods such as class activation mapping [39], and layer-wise relevance propagation [40] including patch-based DL using a deep belief network [41] to separate out the localized features from the predictions. Thus, these methods help to identify the regions of input images that may influence a model’s prediction; however, these local features need not necessarily correspond to the disease-pathological grades.

## Conclusion

In this paper, we propose a hybrid CNN-LSTM method for the classification of histopathological breast cancer images. To increase the robustness of the classifier, we use data augmentation. Deep convolutional features were extracted using ResNet50, InceptionV3, CNN pre-trained on ImageNet and applied LSTM RNN model for classification and compared with three different optimizers and found Adam to be the best optimizer without model overfitting. To our knowledge, the results for both the binary and multi-class classifiers are superior to the state-of-the-art methods. The proposed method is feasible in the classification of other cancers as well as diseases.

## Supplementary Information

**Addiitonal file 1.** Supplementary figures.

## Data Availability

The datasets used and/or analyzed during the current study are available from the corresponding author on reasonable request.
