# Supplementary material for: Classification of benign and malignant subtypes of breast cancer histopathology imaging using hybrid CNN-LSTM based transfer learning
Source: BMC Med Imaging. 2023 Jan 30;23:19. doi: 10.1186/s12880-023-00964-0 (PMC9885590; doi:10.1186/s12880-023-00964-0)
Supplement: Supplementary file 1 — Addiitonal file 1. Supplementary figures. [file 12880_2023_964_MOESM1_ESM.docx]

**Classification of Benign and Malignant Subtypes of Breast Cancer Histopathology Imaging using Hybrid CNN-LSTM based Transfer Learning**

**Supplementary Information**Supplementary data for binary classifier accuracy-loss plots (Fig S1, Fig S2), binary class PR curve plots (Fig S5, Fig S6), binary class ROC curve plots (Fig S7, Fig S8), and multi-class classifier accuracy-loss plots (Fig S3, Fig S4), multi-class PR curve plots (Fig S9, Fig S10), and multi-class ROC curve plots (Fig S11, Fig S12) for RMSprop and SGD optimizers respectively.

**Supplementary Data**

**Fig S1 shows the binary classifier accuracy and error loss plots for (a) 40x (top left), (b) 100x (top right), (c) 200x (bottom-left), and (d) 400x (bottom right) panels respectively for RMSprop optimizer.**


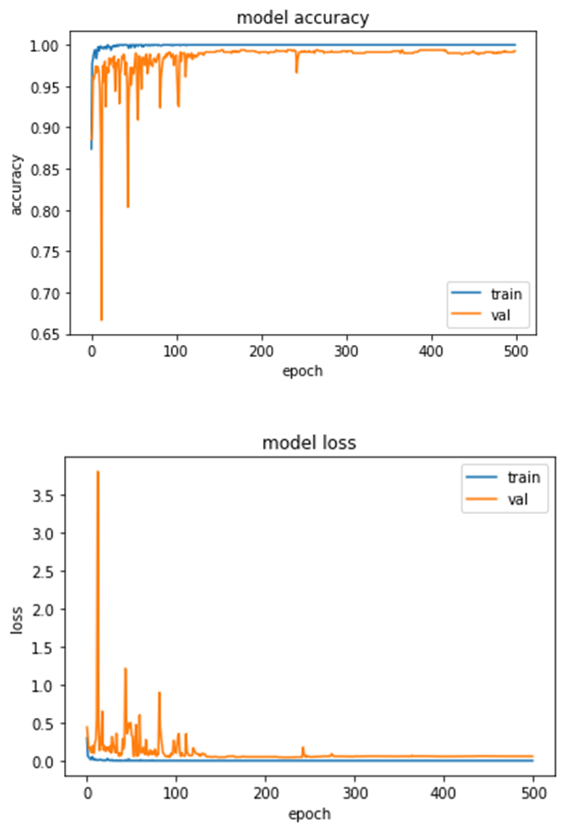

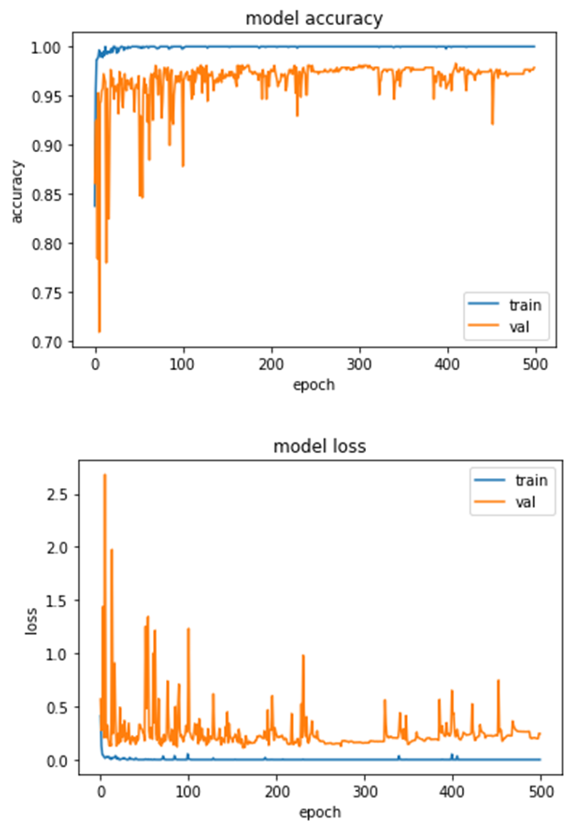


**a**

**b**

**
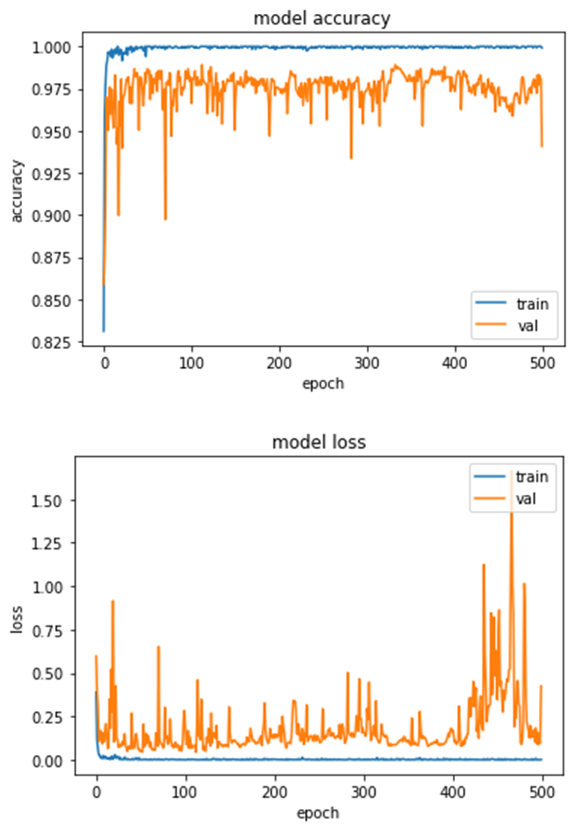

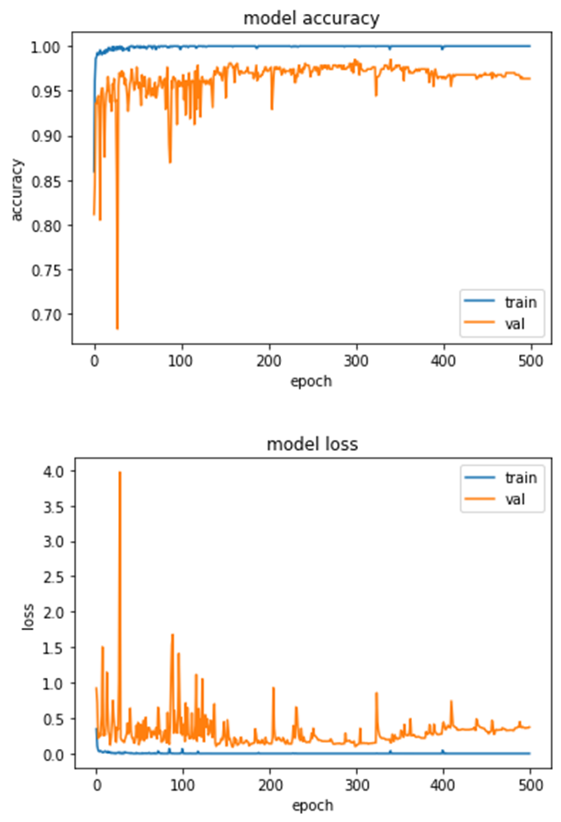
**

**c**

**d**

**Fig S2 shows the binary classifier accuracy and error loss plots for a) 40x (top left), (b) 100x (top right), (c) 200x (bottom-left), and (d) 400x (bottom right) panels respectively for SGD optimizer.**

**
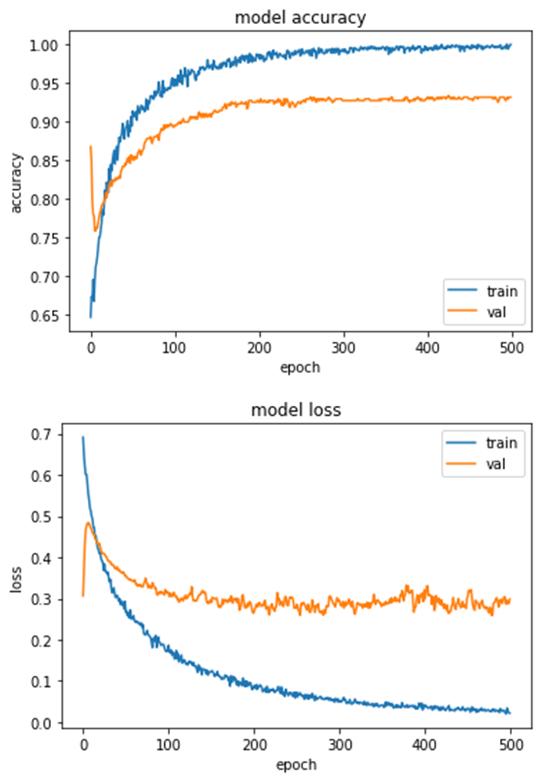

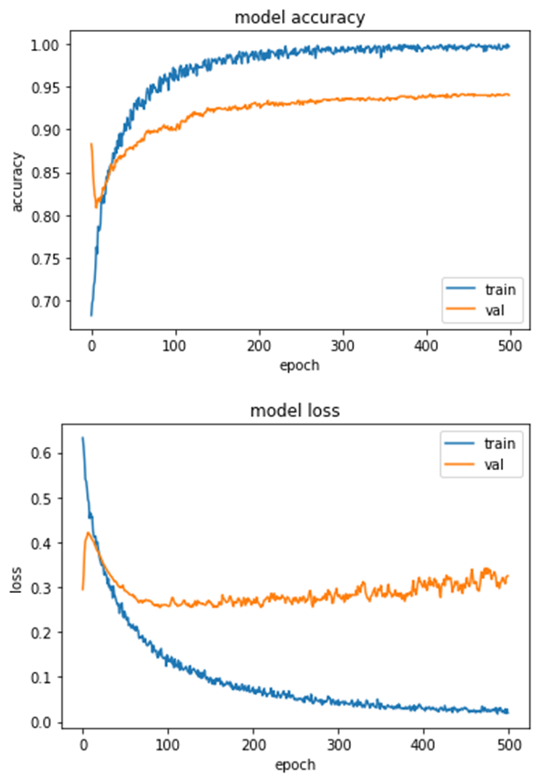
**

**a**

**b**

**
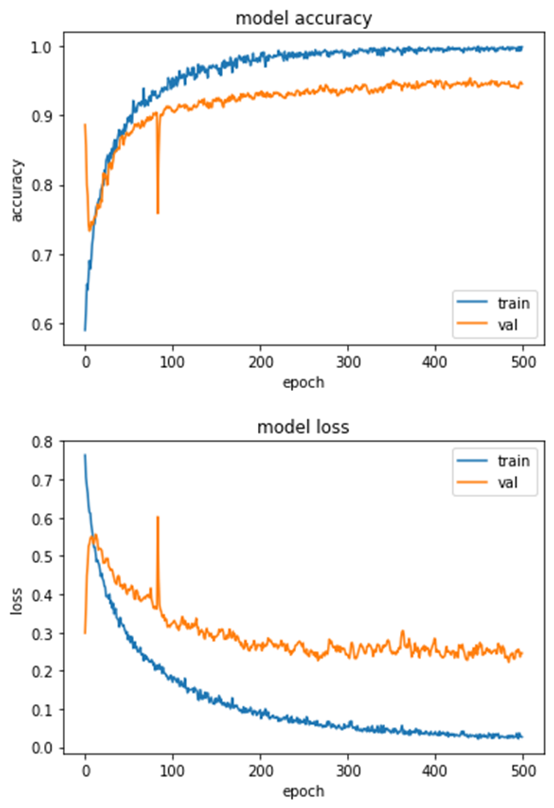

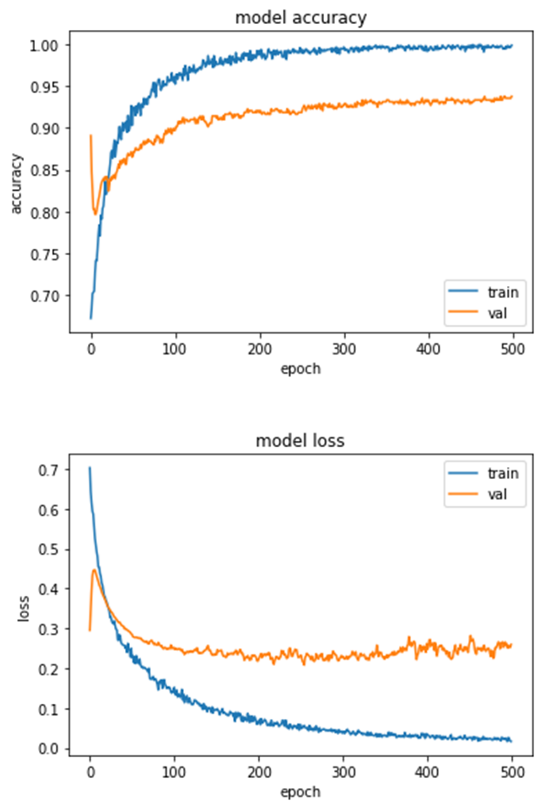
**

**c**

**d**

**Fig S3 shows the multi-classifier accuracy and error loss plots for a) 40x (top left), (b) 100x (top right), (c) 200x (bottom-left), and (d) 400x (bottom right) panels respectively for RMSprop optimizer**

**
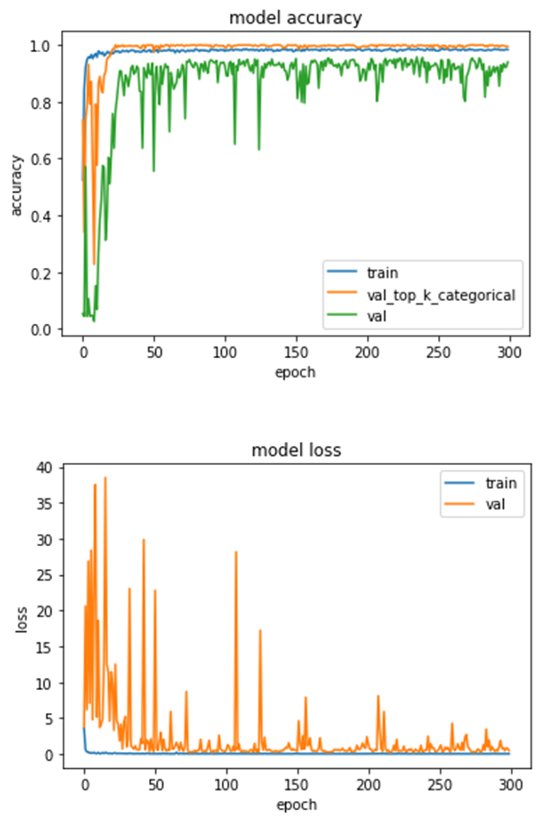

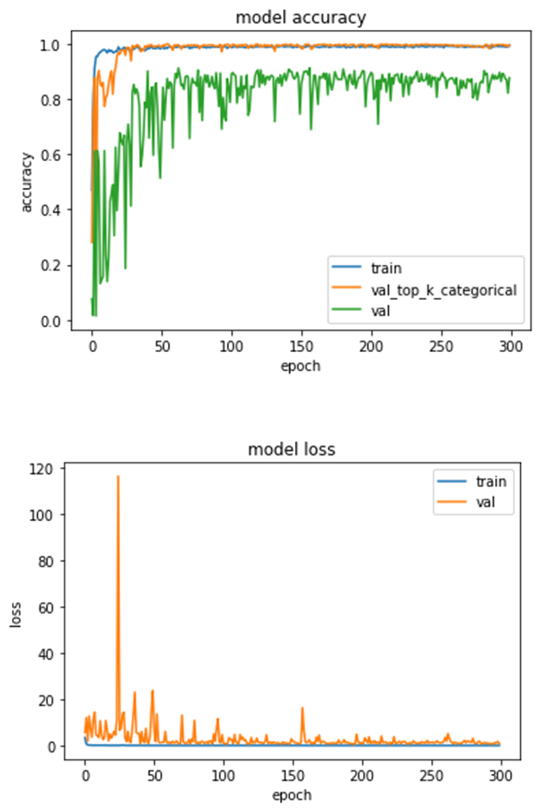
**

**a**

**b**

**c**

**d**

**
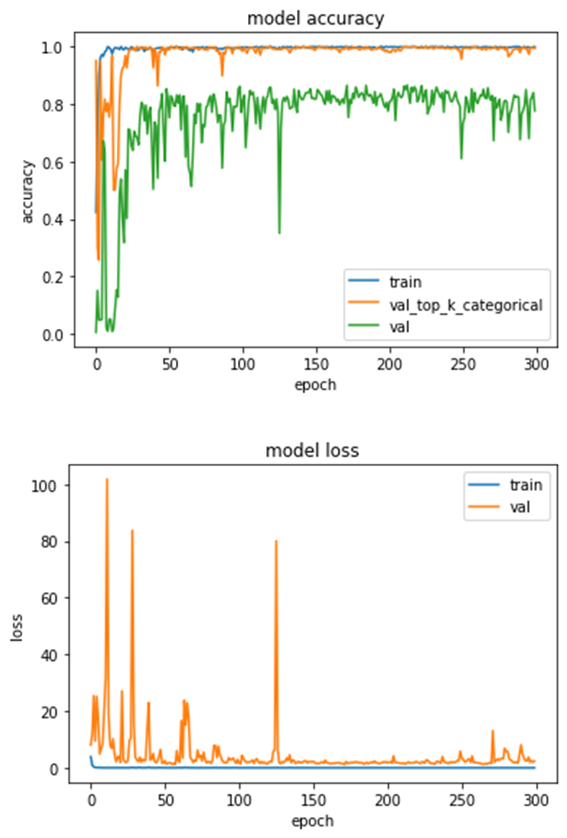

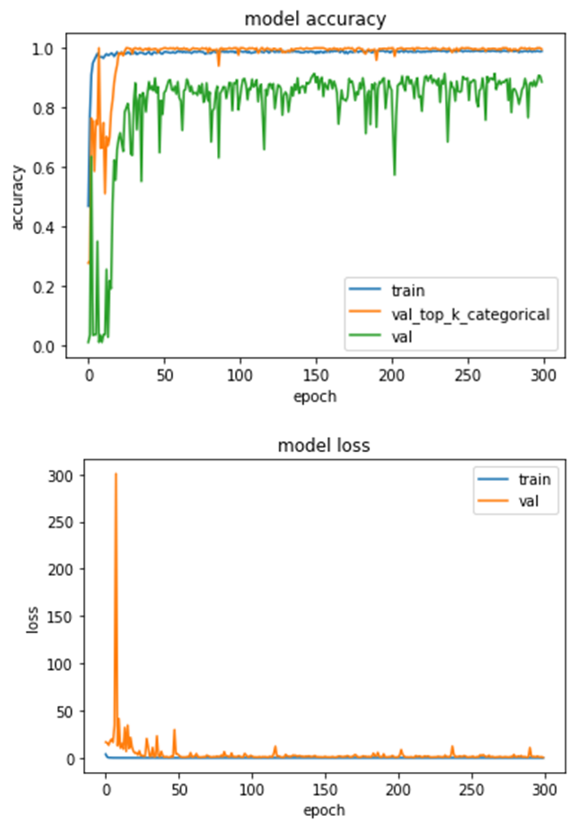
**


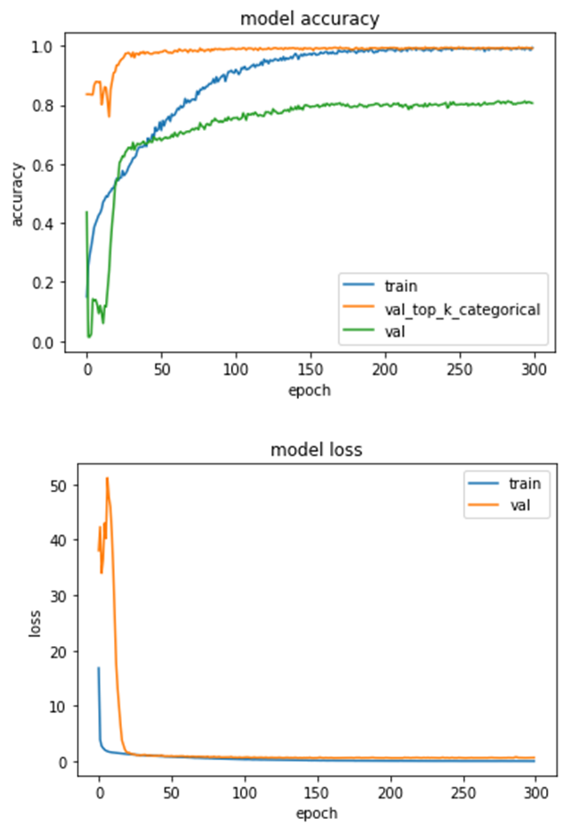

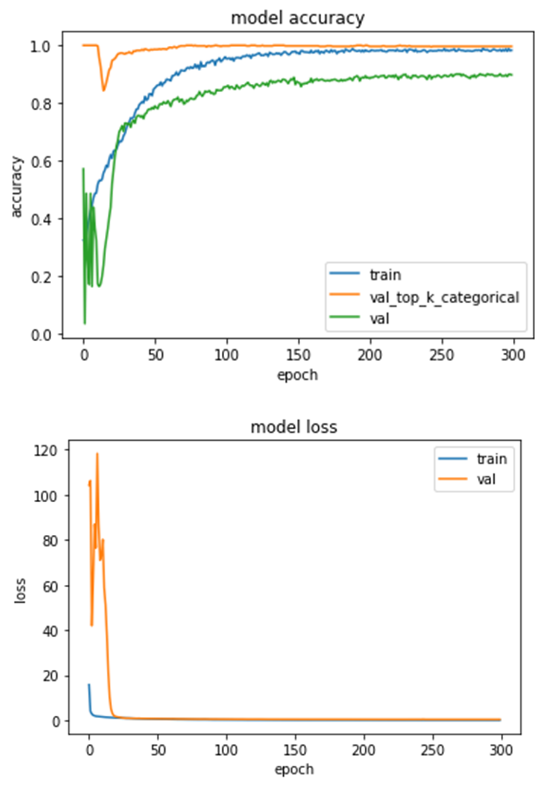
**Fig S4 shows the multi-class classifier accuracy and error loss plots for a) 40x (top left), (b) 100x (top right), (c) 200x (bottom-left), and (d) 400x (bottom right) panels respectively for SGD optimizer**

**b**

**a**

**
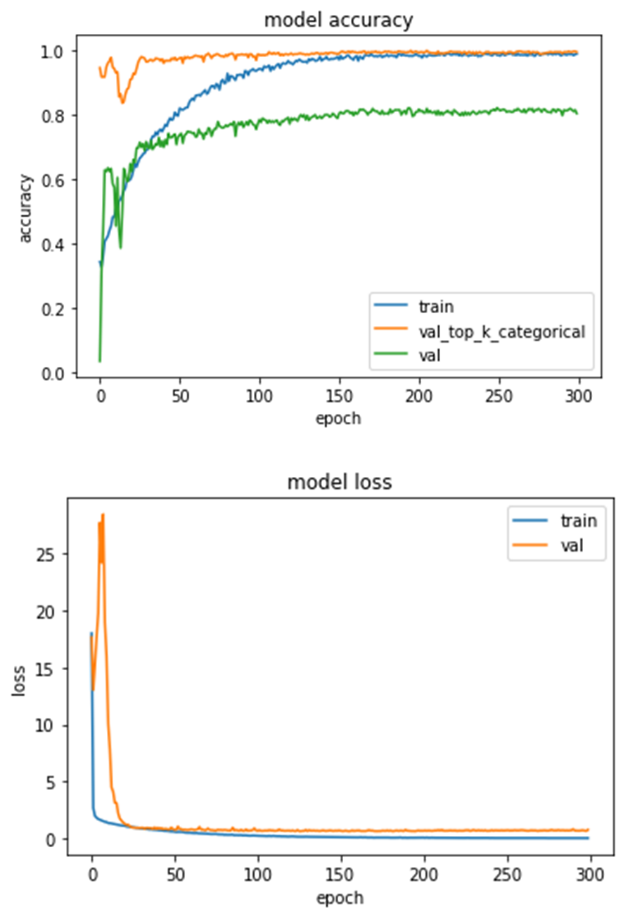

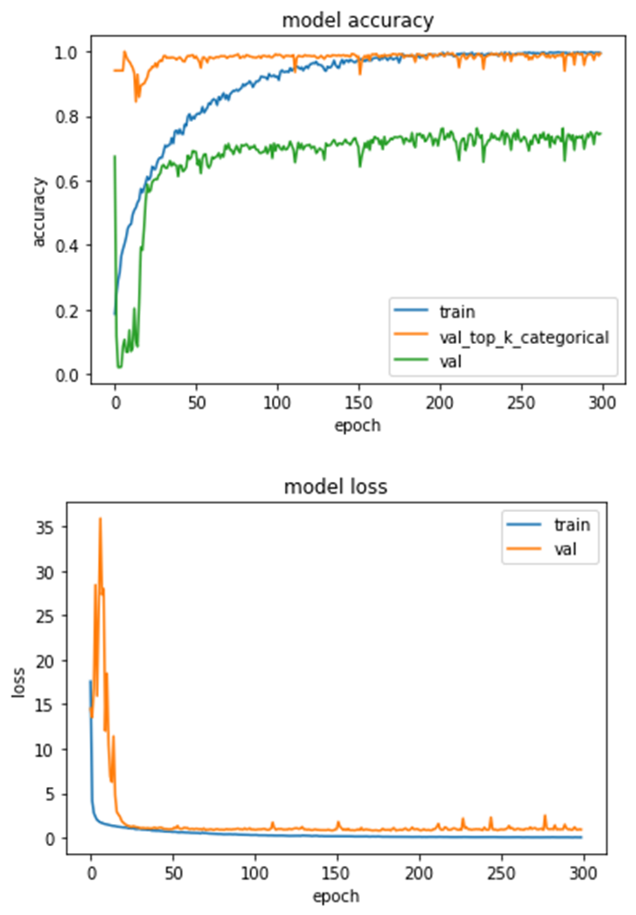
**

**c**

**d**

**Fig S5 shows the binary classifier precision-recall curves for a) 40x (top left), (b) 100x (top right), (c) 200x (bottom-left), and (d) 400x (bottom right) panels respectively for RMSprop optimizer.**

**
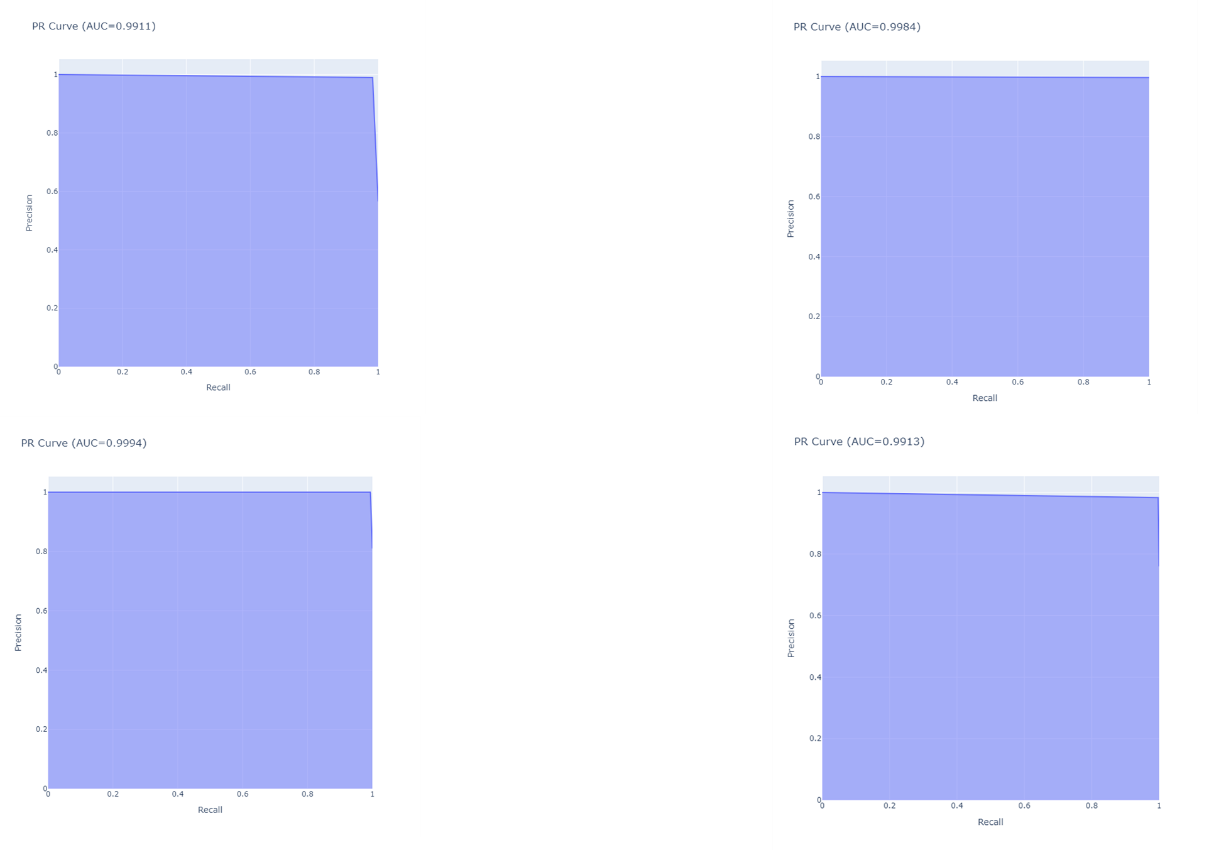
**

**a**

**b**

**c**

**d**


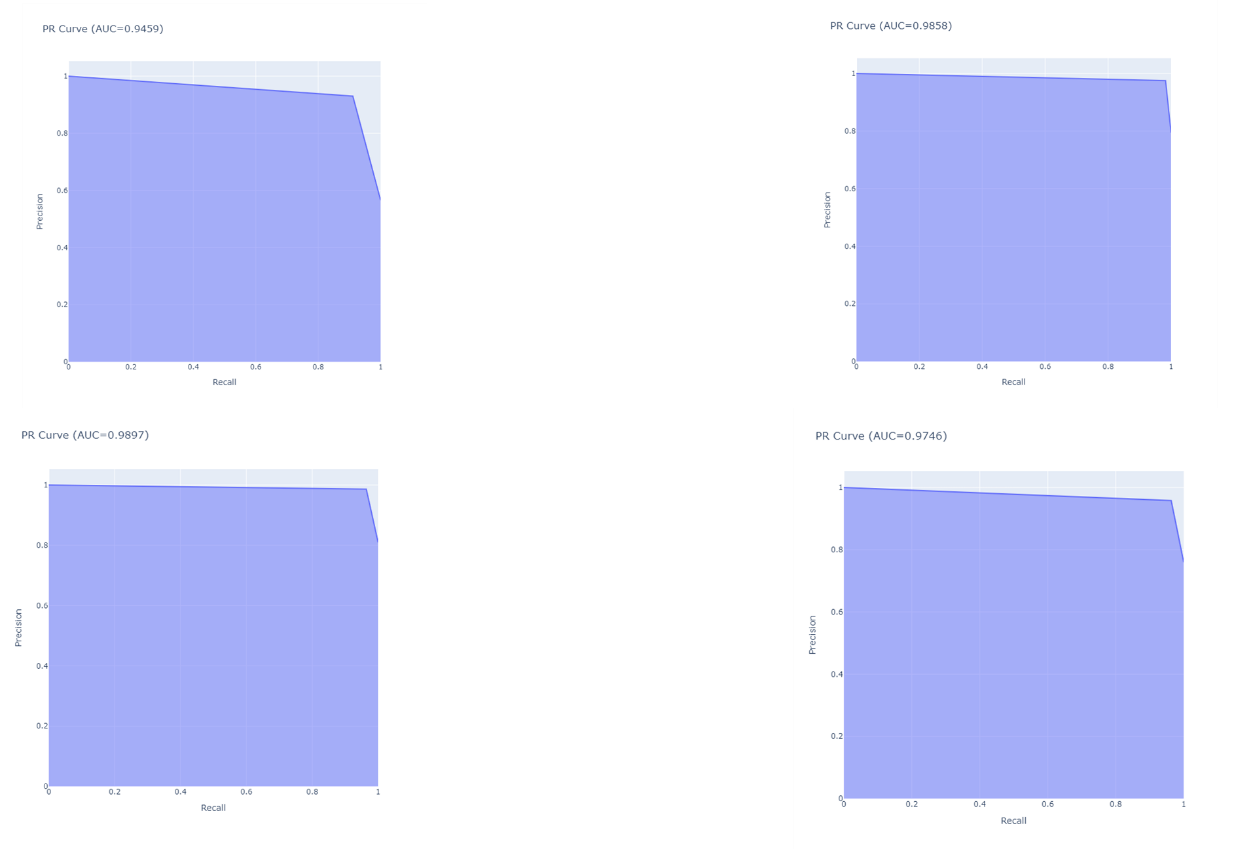
**Fig S6 shows the binary classifier precision-recall curves for a) 40x (top left), (b) 100x (top right), (c) 200x (bottom-left), and (d) 400x (bottom right) panels respectively for SGD optimizer.**

**c**

**d**

**a**

**b**

**c**

**d**


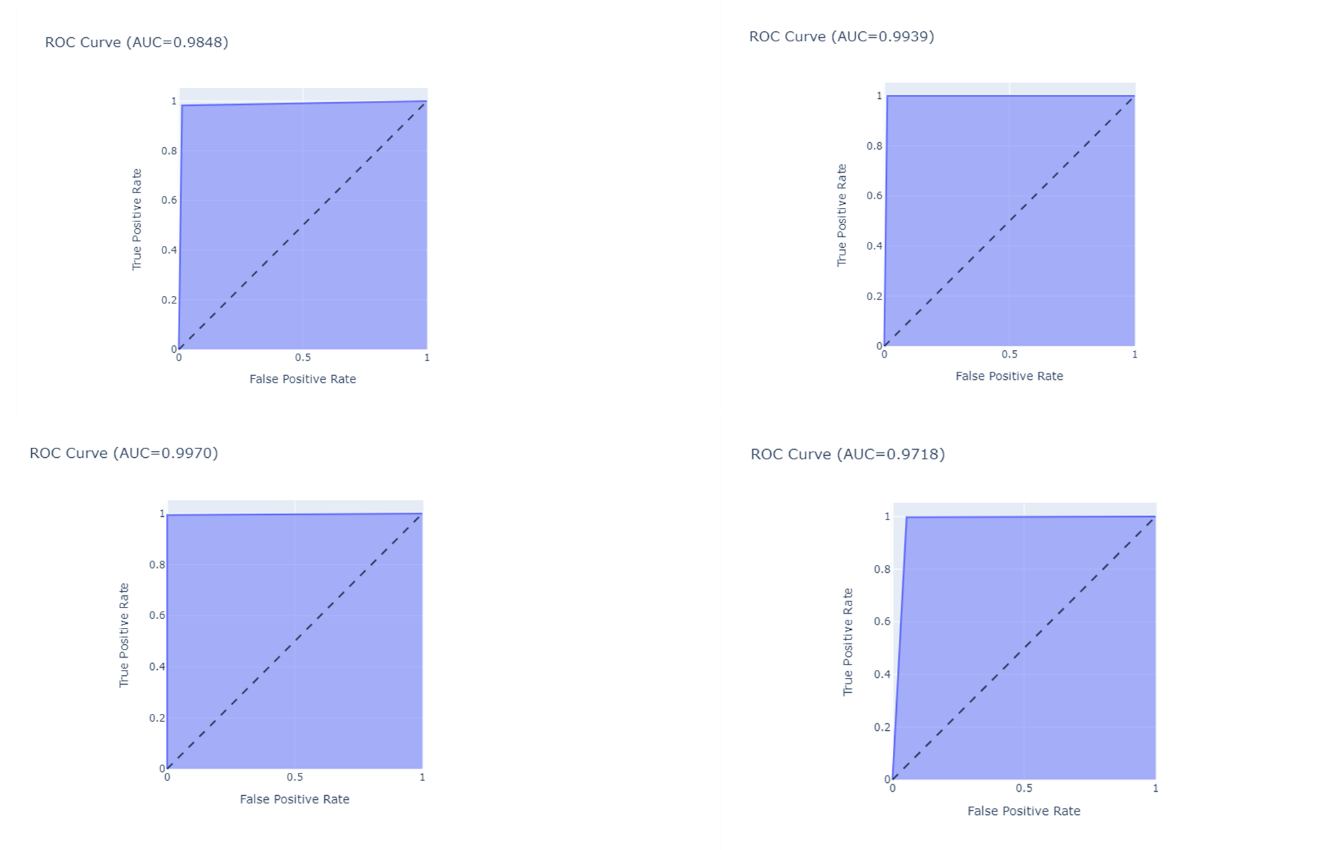
**Fig S7 shows the binary classifier ROC curves for a) 40x (top left), (b) 100x (top right), (c) 200x (bottom-left), and (d) 400x (bottom right) panels respectively for RMSprop optimizer**

**a**

**b**

**c**

**d**

**Fig S8 shows the binary classifier ROC curves for a) 40x (top left), (b) 100x (top right), (c) 200x (bottom-left), and (d) 400x (bottom right) panels respectively for SGD optimizer.**

**a**

**c**

**
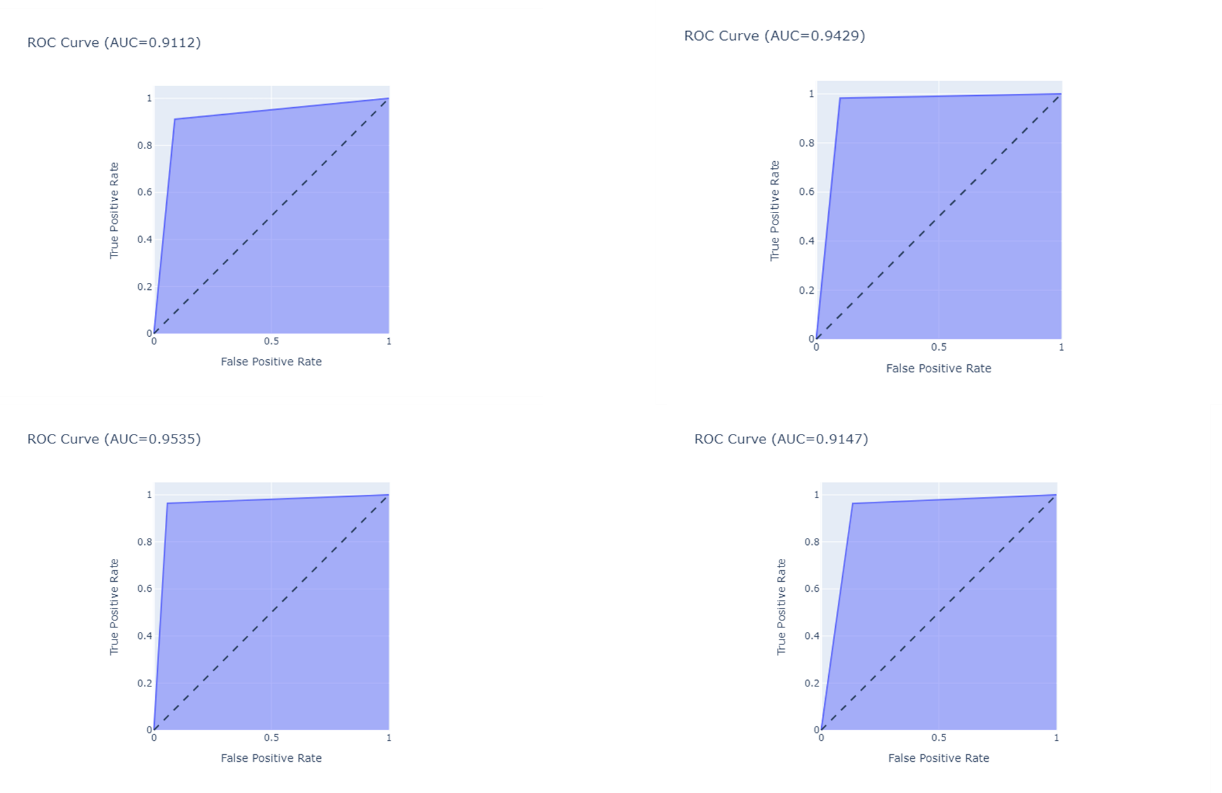
**

**b**

**d**


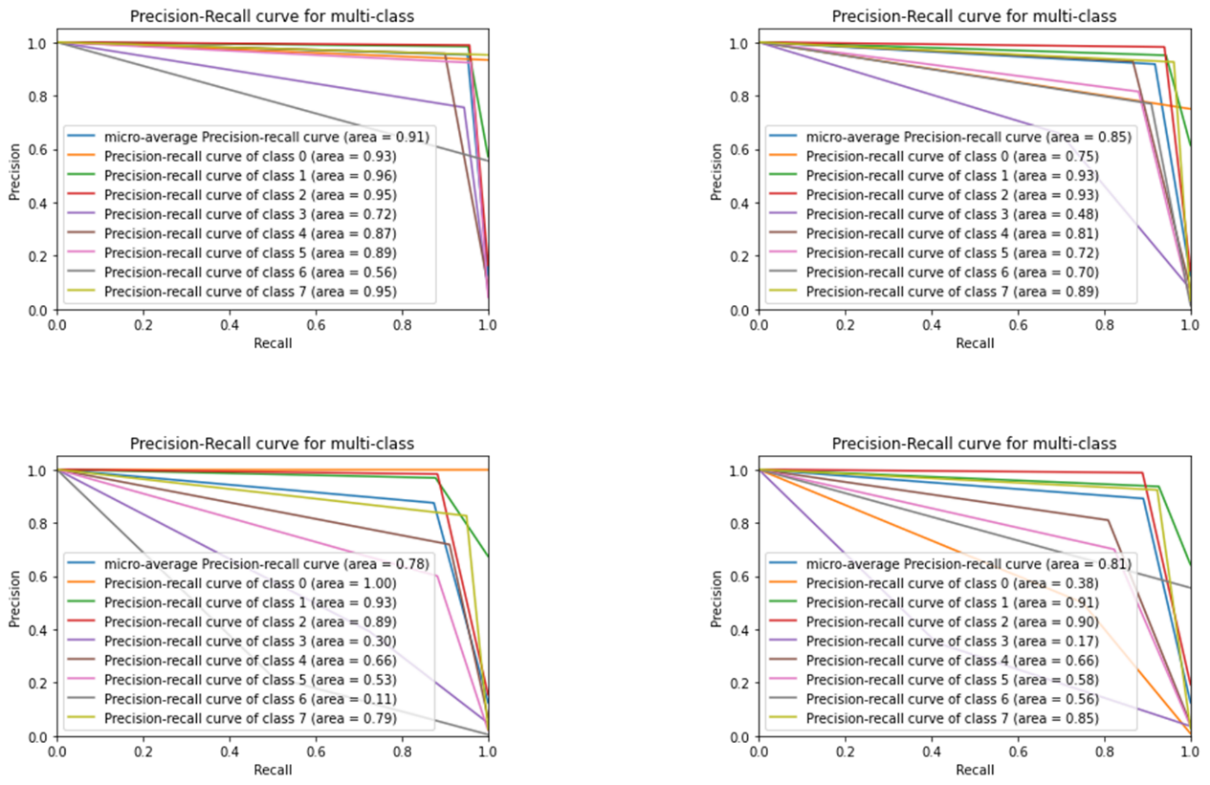
**Fig S9 shows the multi-class classifier precision-recall curves for a) 40x (top left), (b) 100x (top right), (c) 200x (bottom-left), and (d) 400x (bottom right) panels respectively for RMSprop optimizer.**

**a**

**b**

**c**

**d**


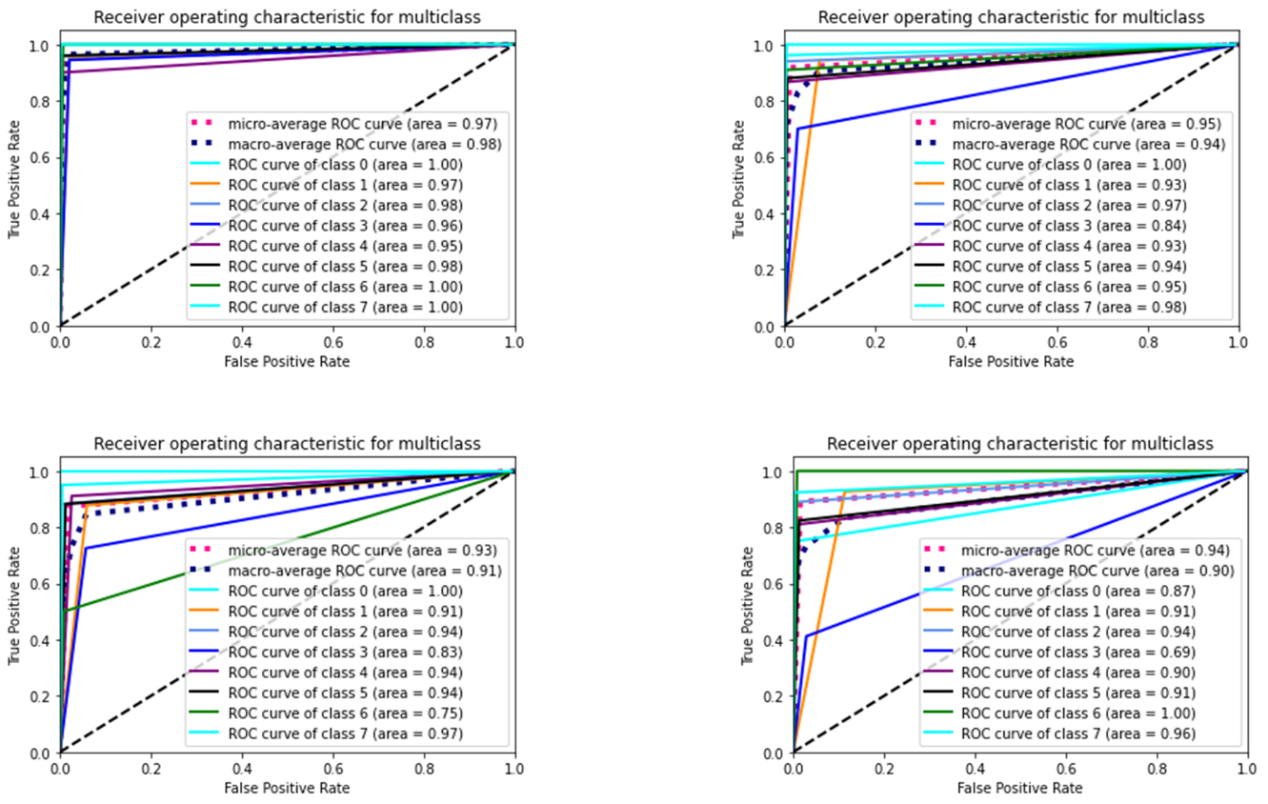
**Fig S10 shows the multi-class classifier precision-recall curves for a) 40x (top left), (b) 100x (top right), (c) 200x (bottom-left), and (d) 400x (bottom right) panels respectively for SGD optimizer.**

**a**

**b**

**d**

**c**

**Fig S11 shows the multi-class classifier ROC curves for a) 40x (top left), (b) 100x (top right), (c) 200x (bottom-left), and (d) 400x (bottom right) panels respectively for RMSprop optimizer.**

**
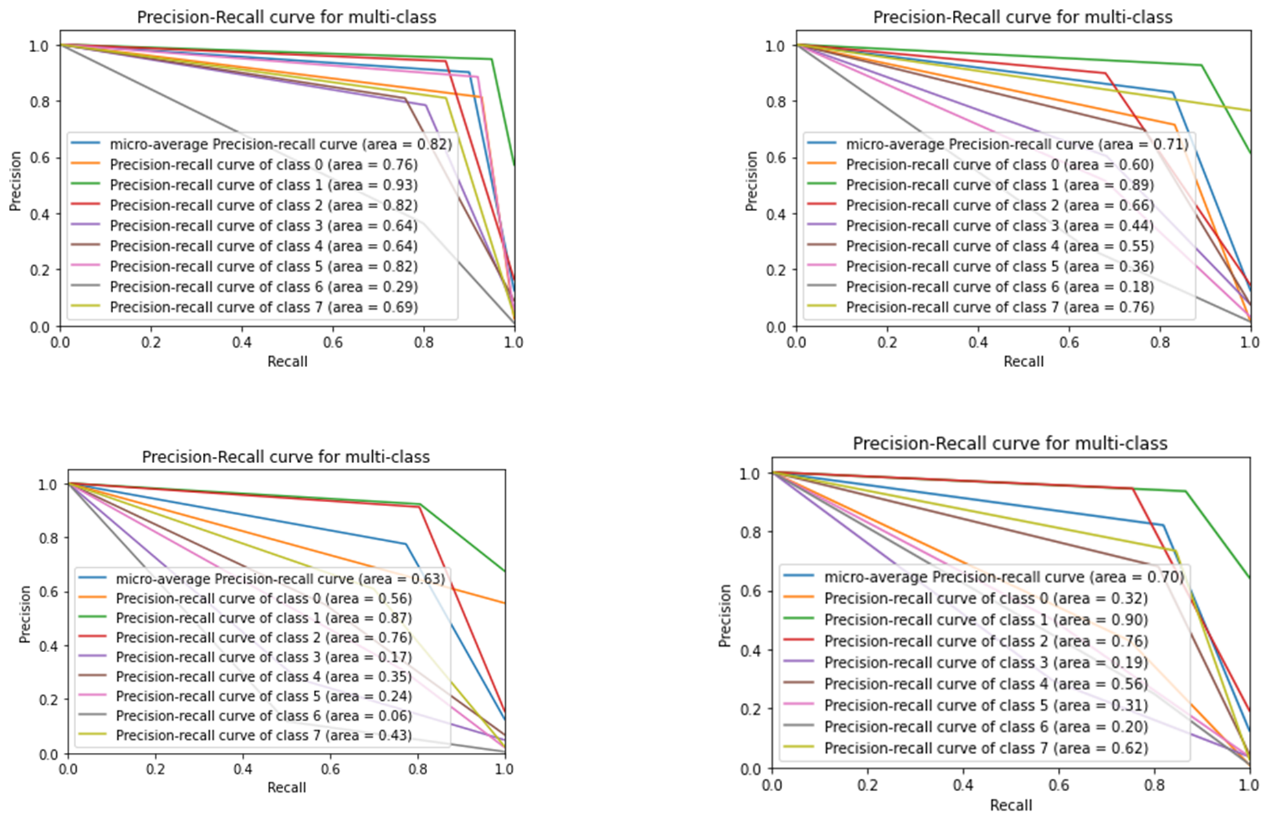
**

**b**

**a**

**c**

**d**


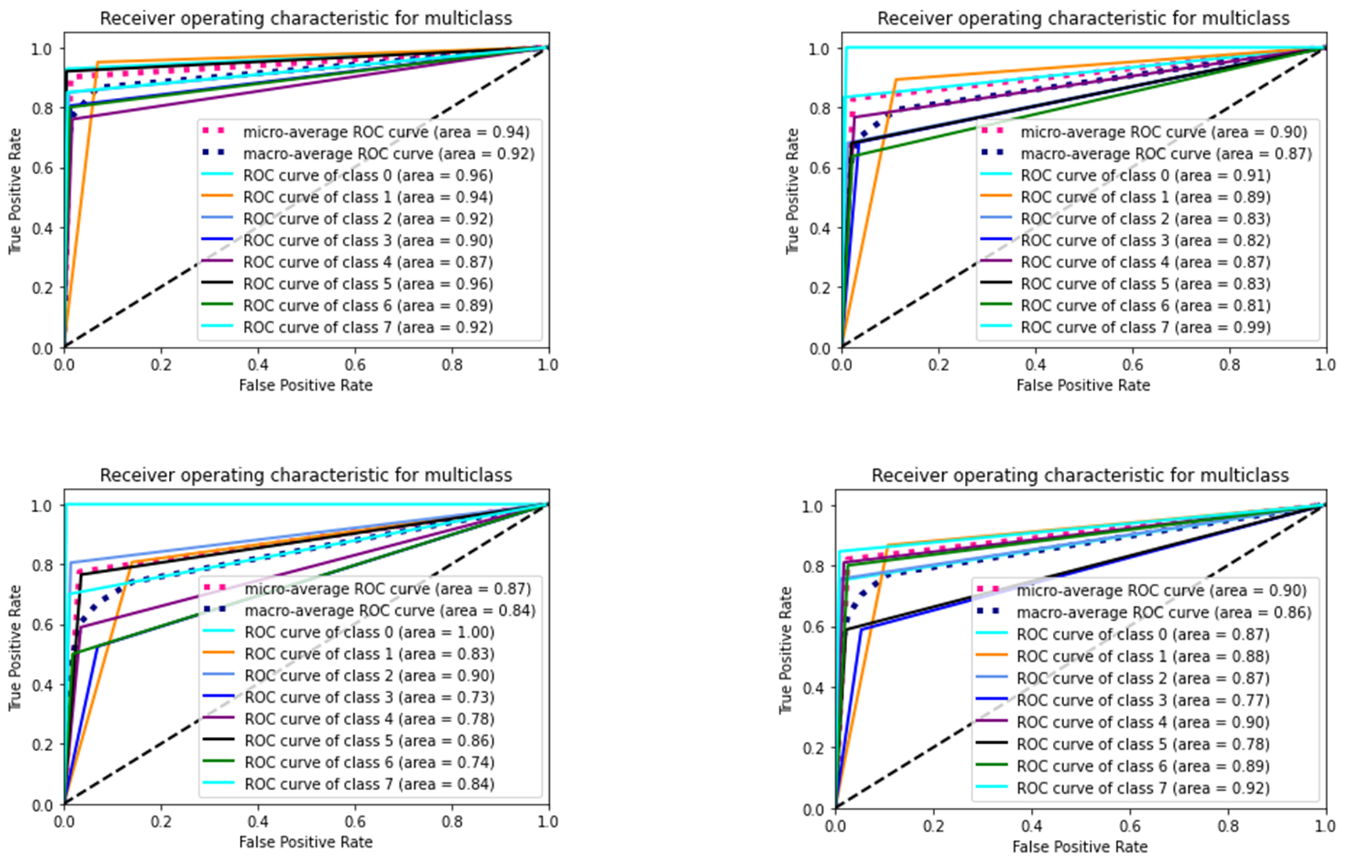
**Fig S12 shows the multi-class classifier ROC curves for a) 40x (top left), (b) 100x (top right), (c) 200x (bottom-left), and (d) 400x (bottom right) panels respectively for SGD optimizer.**

**b**

**d**

**c**

**a**
